# cGAMP the travelling messenger

**DOI:** 10.3389/fimmu.2023.1150705

**Published:** 2023-05-23

**Authors:** Henry T. W. Blest, Lise Chauveau

**Affiliations:** ^1^ Medical Research Council Human Immunology Unit, Medical Research Council Weatherall Institute of Molecular Medicine, Radcliffe Department of Medicine, University of Oxford, Oxford, United Kingdom; ^2^ Institut de Recherche en Infectiologie de Montpellier (IRIM) - CNRS UMR 9004, Université de Montpellier, Montpellier, France

**Keywords:** cGAMP, cGAMP transport, cGAS/STING pathway, vaccine design, cancer immunotherapy, cGAMP conduits

## Abstract

2’3’-cGAMP is a key molecule in the cGAS-STING pathway. This cyclic dinucleotide is produced by the cytosolic DNA sensor cGAS in response to the presence of aberrant dsDNA in the cytoplasm which is associated with microbial invasion or cellular damage. 2’3’-cGAMP acts as a second messenger and activates STING, the central hub of DNA sensing, to induce type-I interferons and pro-inflammatory cytokines necessary for responses against infection, cancer or cellular stress. Classically, detection of pathogens or danger by pattern recognition receptors (PRR) was thought to signal and induce the production of interferon and pro-inflammatory cytokines in the cell where sensing occurred. These interferon and cytokines then signal in both an autocrine and paracrine manner to induce responses in neighboring cells. Deviating from this dogma, recent studies have identified multiple mechanisms by which 2’3’-cGAMP can travel to neighboring cells where it activates STING independent of DNA sensing by cGAS. This observation is of great importance, as the cGAS-STING pathway is involved in immune responses against microbial invaders and cancer while its dysregulation drives the pathology of a wide range of inflammatory diseases to which antagonists have been elusive. In this review, we describe the fast-paced discoveries of the mechanisms by which 2’3’-cGAMP can be transported. We further highlight the diseases where they are important and detail how this change in perspective can be applied to vaccine design, cancer immunotherapies and treatment of cGAS-STING associated disease.

## 1 Introduction

**Figure 1:**
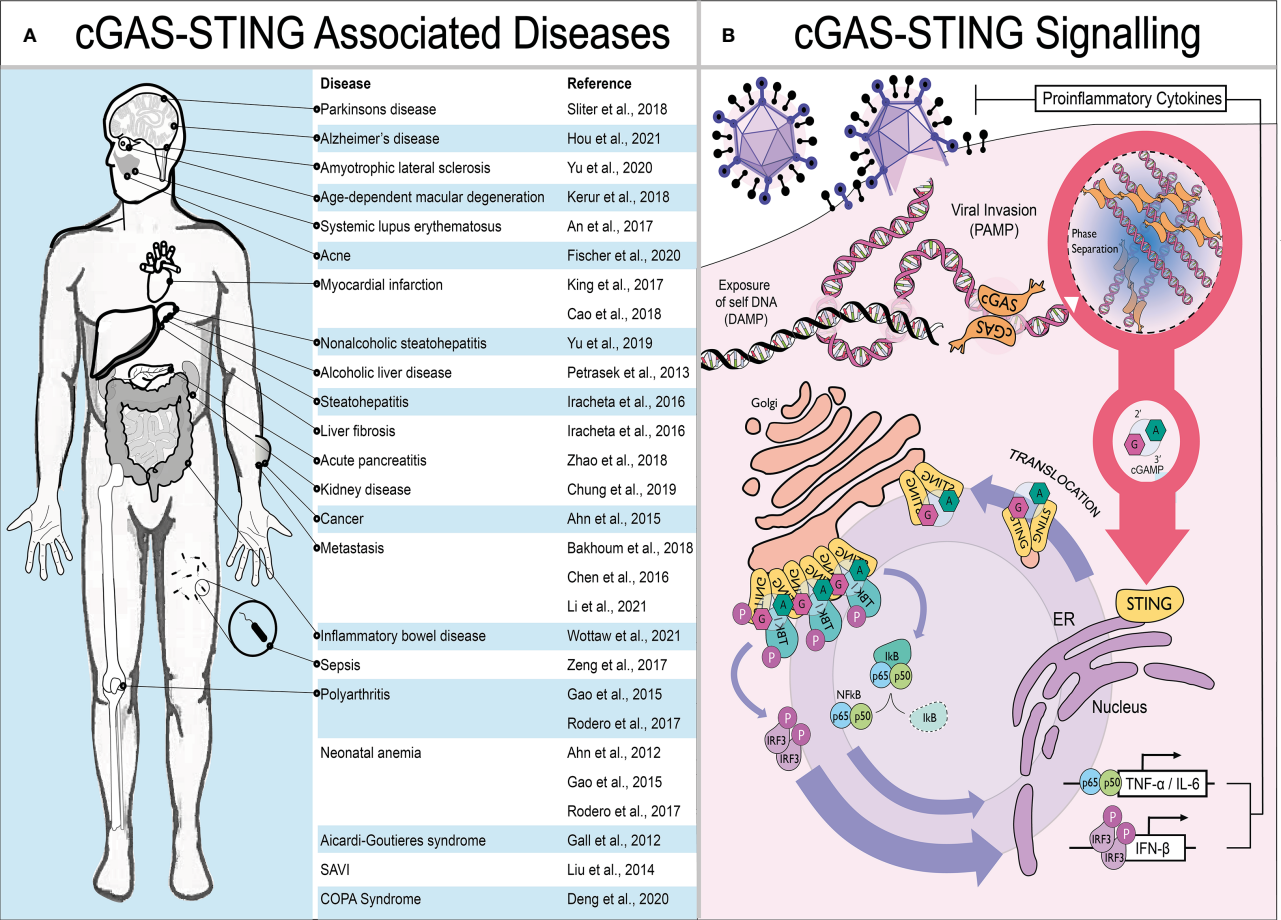
The cGAS-STING pathway and disease. **(A)** Schematic showing diseases where STING signaling is thought to contribute to pathology. **(B)** The presence of mislocalised or pathogen associated dsDNA in the cytoplasm is detected by cGAS. Upon DNA binding, cGAS undergoes phase separation and generates the cyclic dinucleotide 2’3’-cGAMP required by the central hub of DNA sensing – STING – to translocate from the ER towards ERGIC. Here, STING recruits TBK1 to phosphorylate the master transcriptional regulators of innate immune activation - IRF3 and NF-κB. This results in the production of proinflammatory cytokines, primarily type I IFNs but also IL-6 and TNF-α.

Cells utilize an arsenal of pattern recognition receptors (PRRs) which recognize structures within invading microbes termed pathogen associated molecular patterns (PAMPs). Viral genomic nucleic acids are a preeminent PAMP during infection which trigger PRR activation, leading to the production of type-I interferons (IFN) and proinflammatory cytokines by infected cells. IFN then acts in both an autocrine and paracrine manner to induce the expression of interferon-stimulated genes (ISGs) in neighboring cells. These ISGs act as restriction factors in bystander cells to limit pathogen replication and spread. Alongside the direct antimicrobial activity of ISGs, the cytokines produced are also crucial to initiate adaptive immune responses. Since its discovery in 2013, the PRR detecting cytosolic double-stranded DNA (dsDNA) - cyclic guanosine-monophosphate-adenosine-monophosphate synthase (cGAS) - has been a focal point within the field. cGAS detects dsDNA indiscriminate of whether it is host or pathogen derived. Instead of detecting structural differences between pathogen and host like classical PRRs (e.g. TLR9), it identifies the presence of dsDNA in the cytosol as a danger signal. Therefore, exposure of “self” dsDNA due to cellular disruption can also be detected and is a known damage associated molecular pattern (DAMP) that can lead to inflammation in sterile settings. For example, the rare but debilitating interferonopathies - Aicardi-Goutières syndrome (AGS), STING associated vasculopathy with onset in infancy (SAVI) and COPA Syndrome - can be caused by chronic activation of the cGAS-STING pathway (1–3). Moreover, in a wide range of more prevalent autoimmune and inflammatory disorders such as systemic lupus erythematosus, polyarthritis and Parkinson’s disease, STING signaling is thought to contribute towards pathology (4–6). These diseases are summarized in **Figure 1A**. The cGAS-STING pathway is therefore of great therapeutic interest: agonists are sought after for use as adjuvant in vaccines or for cancer immunotherapy; while antagonists could be developed as treatment for inflammatory or auto-immune diseases.

Mechanistically, when cGAS binds to dsDNA, its N-terminal domain polymerises, inducing phase separation (a phenomenon where liquids separate from each other forming distinct membrane-less compartments), and it produces the small cyclic di-nucleotide 2’,3’-cyclic-GMP-AMP (hereafter called cGAMP). cGAMP then acts as a second messenger and binds to the central hub of DNA sensing - STING - which translocates from the endoplasmic reticulum (ER) to the Golgi, where it recruits TANK-binding kinase 1 (TBK1). TBK1, in turn, activates the master regulators of innate immune activation: IRF3 and NFkB. IRF3 induces production of type-I IFN while NFkB drives expression of pro-inflammatory cytokines most notably IL-6 and TNF-α (**Figure 1B**) (7).

Studies published over the last decade challenged the classical model of PRR signaling. Rather than cGAS detecting dsDNA and inducing IFN and cytokine production in the cell where sensing occurred, the second messenger cGAMP can travel to neighboring cells where it activates STING and production of IFN and cytokines without the need for a PRR. Over the last decade, several studies have now described multiple mechanisms by which cGAMP can move between cells. It can travel freely through GAP junctions in juxtaposed cells (8). Moreover, viral particles have been shown to incorporate cGAMP derived from the viral producing cells, thereby transmitting cGAMP to the next infected cell (9, 10). Both of these mechanisms effectively link the cytosol of one cell to the next and so cGAMP remains topologically contained within a compartment derived from the cell cytosol. More recently a role for cGAMP in the extracellular space has been considered. Exogenous cGAMP, and other similar cyclic dinucleotides (CDNs) produced by bacteria as signaling molecules, were known to trigger IFN-β production by cells *in vitro* and reduce tumor growth by direct injection *in vivo* (11–16). However, it remained unclear how these CDNs entered the cytosol to activate STING. This changed between 2019 and 2021 when, in quick succession, five membrane transport proteins were shown to provide a route for CDNs to cross the cell membrane - SLC19A1, SLC46A2, P2X7, LRRC8A:C/E and ABCC1 (12, 13, 17–21). In addition, the antimicrobial defense peptide LL-37 has been reported to bind and chaperone cGAMP across the cell membrane (22). These proteins are separated into channels (SLC19A1, SLC46A2 and LRRC8A:C/E), transporters (ABCC1) and pores (P2X7 and LL-37) depending on their mode of action. For simplicity, we will collectively refer to them as cGAMP conduits.

**Figure 2:**
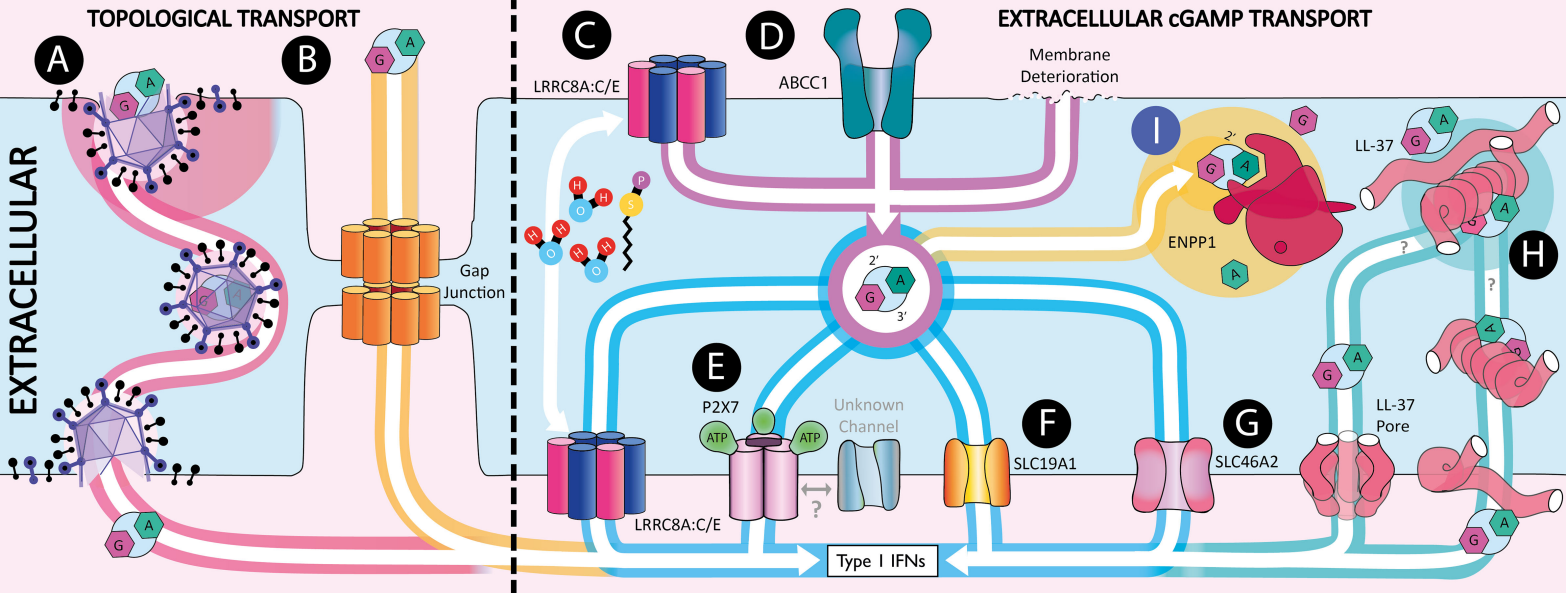
Extracellular 2’3’-cGAMP transport. Schematic showing the mechanisms by which 2’3’-cGAMP can be transferred between cells. **(A)** Within viral particles. **(B)** Through gap junctions. **(C)** Import and export through LRRC8A:C heterodimers which are activated by hypotonicity and Sphingosine-1-phosphate (S1P). **(D)** Export through ABCC1. **(E)** Transport through the ATP gated pore P2X7. **(F)** Import through SLC19A1. **(G)** Import through SLC46A2. **(H)** 2’3’-cGAMP shuttling by LL-37. **(I)** 2’3’-cGAMP hydrolysis by ENPP1.

The study of how cGAMP is a second messenger that travels between cells constitutes a new field that has recently exploded. In this review, we detail and discuss the current knowledge of the different mechanisms by which cGAMP is transferred between cells summarized in **Figure 2**. We further highlight the diseases where these mechanisms play a role and the relevance this has to pharmaceutical design suggesting novel targets for innovative therapies.

## 2 Mechanisms of cGAMP inter-cellular signaling

**Figure 3:**
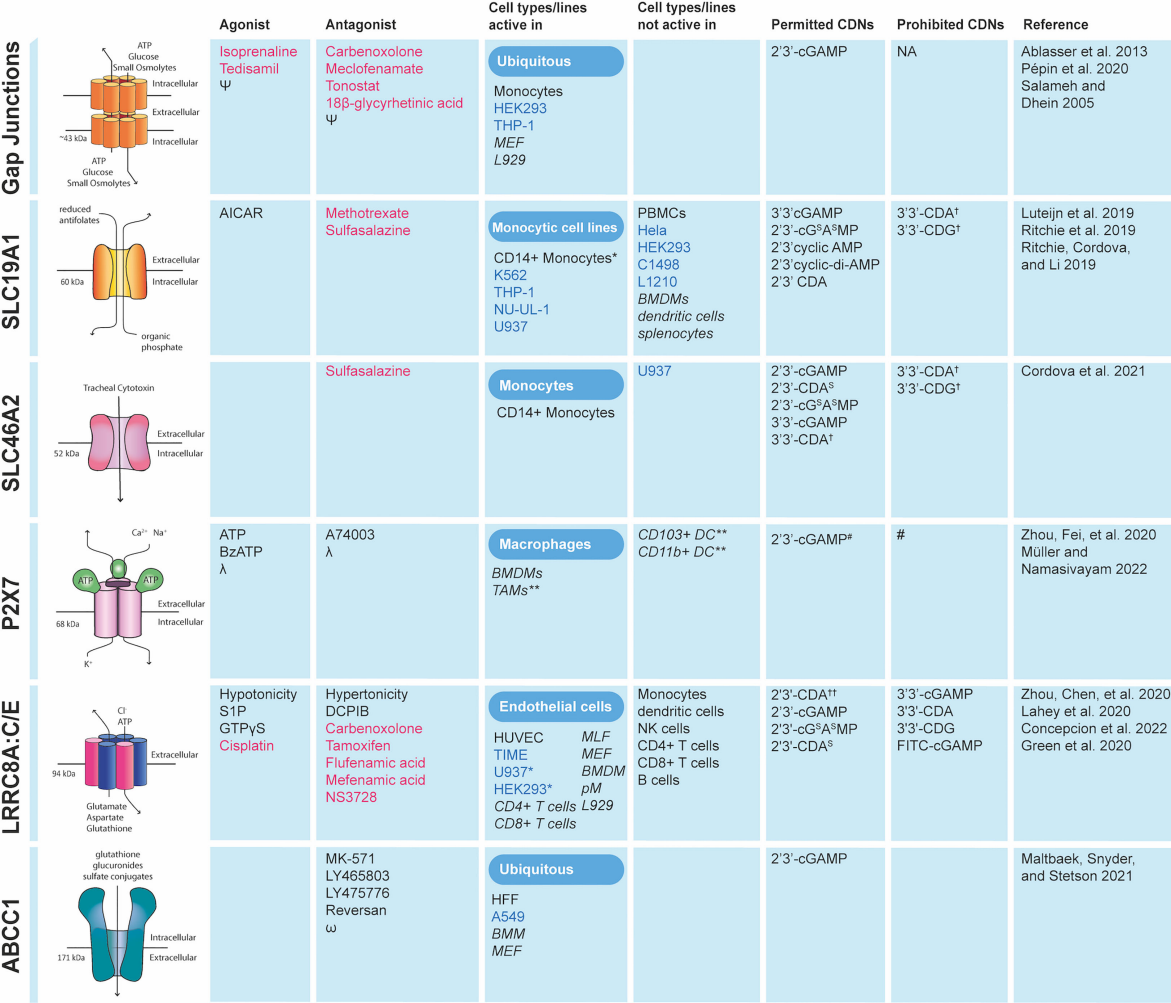
Cell types in which cGAMP conduits function and the CDNs they transport. Data collected for cells types in which conduit deficiency or specific chemical inhibition prevented STING pathway activation in response to exogenous CDNs. FDA approved drugs are shown in red. Primary cells are shown in black and cancer cell lines in blue. Mouse cell lines and conduits tested in mice are shown in italics. ϕ sulfasalazine has been shown not only to inhibit indicated channels but also IFN-β production *Not major importer as knockout or chemical inhibition only marginally reduces CDN mediated responses. **Direct CDN uptake not proven *in vitro* but *in vivo* conduit required for STING pathway activation in presence of exogenous CDNs. †Unclear if imported. †† Minimal effect of transporter on uptake. # - P2X7 forms a large non-selective pore for hydrophilic substances ~0.9 KDa in size therefore although not formally shown it is assumed there is no CDN specificity. Ψ reviewed by Salameh and Dhein (23). λ reviewed by Müller and Namasivayam (24). ω reviewed by Cole (25). MLF, mouse lymphatic fibroblasts; BMM, bone marrow-derived macrophages; MEF, mouse embryonic fibroblasts; HFF, human foreskin fibroblasts; BMDM, bone-marrow-derived macrophages; pM, peritoneal macrophages; TAM, Tumor-associated macrophages; PBMC, peripheral blood mononuclear cells.

In this section, we describe the molecular mechanisms that are responsible for transporting cGAMP between cells. We first take into consideration the role of ENPP1 in extracellular cGAMP signaling, as it is the only described human hydrolase that degrades cGAMP. We then highlight the conduits that allow the export and import of free cGAMP to and from the extracellular space. We term this extracellular cGAMP transport. The known agonists and antagonists of cGAMP conduits as well as the cell types they have been shown to function in are summarized in **Figure 3**. Finally, we describe the two known mechanisms by which cGAMP is transferred between cells in compartments topologically equivalent to the cytosol, thereby protecting it from ENPP1 hydrolysis. We term this topologically contained cGAMP transport.

### 2.1 Extracellular cGAMP transport

#### 2.1.1 ENPP1

To understand the role of extracellular cGAMP, we must first consider ecto-nucleotide pyrophosphatase/phosphodiesterase (ENPP1). ENPP1 is a hydrolase of CDNs and ATP. Studies overexpressing ENPP1 show its action is limited to the extracellular space despite being present within the lumen of the endoplasmic reticulum (ER) (26). Structurally, it contains a Ca^2+^ binding domain and chelates two Zn^2+^ ions into its catalytic site. These ions are required for ENPP1 function and supplementation has been shown to enhance hydrolase activity (27). ENPP1’s hydrophobic N-terminus can be cleaved, releasing the now soluble and hydrolysis active enzyme into the surrounding tissue fluid (28). Notably, extracts from WT mouse spleen and liver can completely hydrolyze cGAMP but extracts from *Enpp1*-/- knockout mice show no detectable degradation. This demonstrates that at least in mice, Enpp1 is likely the dominant if not the only cGAMP hydrolase (27). It is unclear if tissues lacking ENPP1 expression, or a yet to be identified regulator of this hydrolase, could allow for extracellular cGAMP accumulation and immune activation. Overall, ENPP1 represents the only known mechanism of cGAMP degradation and its discovery provided the first evidence that cGAMP might have relevance outside of the cell. Its identification gave the rationale to search for the cGAMP conduits described below.

#### 2.1.2 SLC19A1

The first cGAMP conduit to be reported was reduced folate carrier 1 (SLC19A1/RFC1). SLC19A1 was initially characterized as an anti-transporter and main importer of reduced folate into cells. This couples the import of reduced/anti folates to the export of inorganic phosphates (29). Folates are essential membrane impermeable B family vitamins required for cell proliferation, tissue repair and development (29). Hence knockout of SLC19A1 in mice is embryonically lethal without continuous folate supplementation (30).

**Figure 4:**
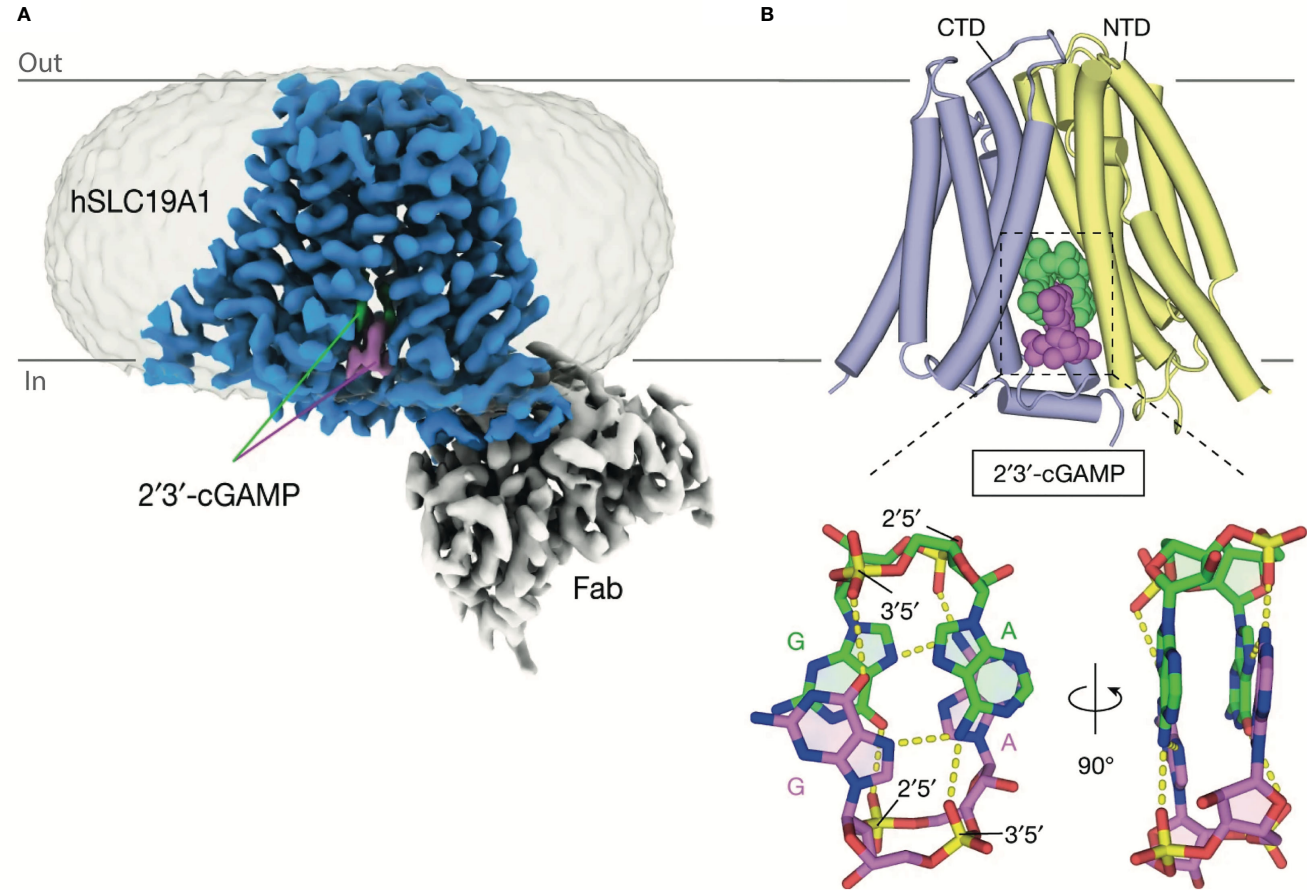
Structure of SLC19A1 in complex with 2’3’-cGAMP. **(A)** Cryo-EM density map of human SLC19A1(blue)-Fab(grey) complex bound to two molecules of 2’3’-cGAMP (green and purple). **(B)** The overall structures of human SLC19A1 bound to 2′3′-cGAMP in the inward-open conformation. The N-terminal domain (NTD) is colored yellow and C-terminal domain (CTD) colored blue. Two bound 2′3′-cGAMP molecules are shown as a space-filling representation and are colored (green and purple). Adapted with permission from Zhang, Zhang (31).

Initially, SLC19A1’s role in cGAMP transport was characterized in the U937 and THP-1 human monocytic cell lines. SLC19A1 knockout was seen to prevent STING activation in response to extracellular cGAMP. Chemical characterization showed that methotrexate, a competitive inhibitor of SLC19A1, similarly prevented STING activation in response to exogenous cGAMP (12, 13). Finally, a direct interaction between cGAMP and SLC19A1 has been shown as SLC19A1 precipitates with cGAMP immobilized on sepharose beads. This interaction is disrupted by free unbound cGAMP or methotrexate (13). More recently the structure of SLC19A1 in complex with cGAMP has been solved showing how cGAMP could move directly through this channel (**Figure 4**) (31). At the time of writing, SLC19A1 is the only cGAMP conduit for which a direct interaction is demonstrated and the exact mechanisms used by other conduits are still under investigation. Although SLC19A1’s function has been observed in human monocytic cell lines (THP1 and U937), only a minimal role in cGAMP uptake has been shown in primary human monocytes (12). This suggests SLC19A1 plays a non-dominant role in monocytic cGAMP import and highlights the caution and need for primary cell data to delineate the cell types in which these channels transport cGAMP *in vivo*.

Other cyclic dinucleotides in addition to 2’3’-cGAMP are imported into cells by SLC19A1, both synthetic (2’3’-RR CDA, 2’3’-cG^S^A^S^MP, 2’3’-CDA, 2’3’-CDA^S^) and bacterially derived CDNs (3’3’-cGAMP). In contrast, some bacterially derived CDNs were imported poorly or not at all (e.g. 3’3’-CDA, 3’3’-CDG) (**Figure 3**). The possibility that SLC19A1 shows specificity to the type of CDNs that are imported is exciting, as the knowledge of the cell types expressing SLC19A1 could allow for cell targeted CDN treatments based on CDN conduit expression profiles (12).

Murine SLC19A1 is not thought to import cGAMP since efficient depletion of *Slc19a1* in mouse cell lines has no effect on cGAMP uptake. Similarly, in murine primary bone marrow derived macrophages and dendritic cells, *Slc19a1* depletion does not reduce IFN-β mRNA levels upon CDN treatment. This suggests another importer is mediating cGAMP uptake in murine cells (13). Therefore, there is a need for a suitable animal model to study this channel’s role in the context of viral infection or cancer *in vivo*. Indeed, without it, it cannot be proven that SLC19A1 functions physiologically to enhance cGAMP driven immune responses, especially as it is possible that the wide variety of natural SLC19A1 substrates may out compete cGAMP for import. Future studies looking at viral antagonism *in vitro* or polymorphisms within patient cohorts will also be key to provide evidence of its role at the physiological level.

#### 2.1.3 SLC46A2

SLC46A2 (TSCOT) is an orphan solute carrier with predominant expression known in the cortical epithelial cells of the murine thymus (32). Its only known substrate is foreign: the tracheal cytoxin (TCT) produced by *Bordetella pertussis* and *Neisseria gonorrhoeae* that enters mammalian cells and drives NOD1 activation and necrosis (33). Therefore, an endogenous role was missing.

SLC46A2’s role in cGAMP transport was initially identified by Cordova et al., who observed it is the dominant cGAMP importer in human monocytes and macrophages (17). This was shown by partial knockdown of SLC46A2, resulting in reduced STING activation in response to extracellular cGAMP in primary monocytes. Additionally, overexpression of human and murine SLC46A2 enhances STING activation in response to extracellular but not electroporated cGAMP. SLC46A2 is a member of the SLC46A solute transporter family, which has two other members: SLC46A1 and SLC46A3. SLC46A1, like SLC19A1, transports folate species into cells. Unlike SLC19A1, it functions optimally at low pH (~5.5) and is predominately responsible for folate uptake in the small intestine (34). SLC46A3, like SLC46A2, is an orphan solute carrier without known function (35). Interestingly, SLC46A1 and SLC46A3 appeared as prominent hits in a previous screen looking to identify cGAMP importers (13). On overexpression, these proteins enhance STING activation in response to extracellular cGAMP. Despite this, effective depletion of SLC46A1 and SLC46A3 caused no or very minor inhibition of STING activation in monocytes. This is likely due to the redundant and highly efficient uptake through SLC46A2 (13, 17). As such SLC46A1 and SLC46A3 are likely able to transport cGAMP but a physiological role is still to be explored. Research into whether SLC46A1 aids STING activation in the gut could answer this as it is highly expressed in the intestines, proximal to CDN producing bacteria and a site of autoimmune disease (35).

Despite murine SLC46A2 being able to transport cGAMP, it is not responsible for uptake in murine macrophages, as SLC46A2 is poorly expressed in murine immune cells (17). SLC46A1 and SLC46A3 are expressed but have also been shown not to be the dominant transporter (17). This suggests there is another mechanism of cGAMP uptake in murine macrophages. Therefore, like with SLC19A1, there are clear differences in SLC46A2 mediated cGAMP uptake between humans and mice.

#### 2.1.4 P2X7

Extracellular ATP functions in the synaptic cleft as a neurotransmitter, but widespread release is a renowned DAMP that coordinates immune responses in various pathological settings (e.g. microbial invasion, cancer and tissue damage) (36). Membrane disintegration of dying or damaged cells is thought to release ATP into the extracellular space. Additionally, channels transport ATP into secretory vesicles or directly into the extracellular space (e.g. VRACs, pannexins, connexins) when triggered by various stimuli (e.g. Ca^2+^ currents, CO_2_, mechanical deformation, hypoxia, heat and osmotic stress) (37). Remarkably, levels of ATP in the tumor microenvironment have been shown to be up to 1000 times greater than that of healthy tissue (38). In mammals, released ATP is detected by two G-Protein coupled P2Y receptors (P2Y1R and P2Y11R) and by all 7 of the P2X ligand gated ion channels (P2X1-7) (39). The P2XRs are mainly expressed in excitable and immune cells, in line with ATP’s role as both a neurotransmitter and DAMP. Notably, P2X7 is expressed in virtually all immune cells (including: macrophages, dendritic cells, granulocytes, T cells, B cells and natural killer cells) and confers resistance to bacterial, parasitic and viral infection (39). Under resting conditions P2X7 supports Na^+^ and Ca^2+^ influx and minimal K^+^ leakage out of the cell. Activation of P2X7 by ATP causes the opening of a non-selective pore for hydrophilic substances of up to ~0.9 KDa and passive K^+^ efflux (40, 41). cGAMP has a molecular weight of ~0.7 kDa, meaning passage through the activated P2X7 pore is feasible (27).

**Figure 5:**
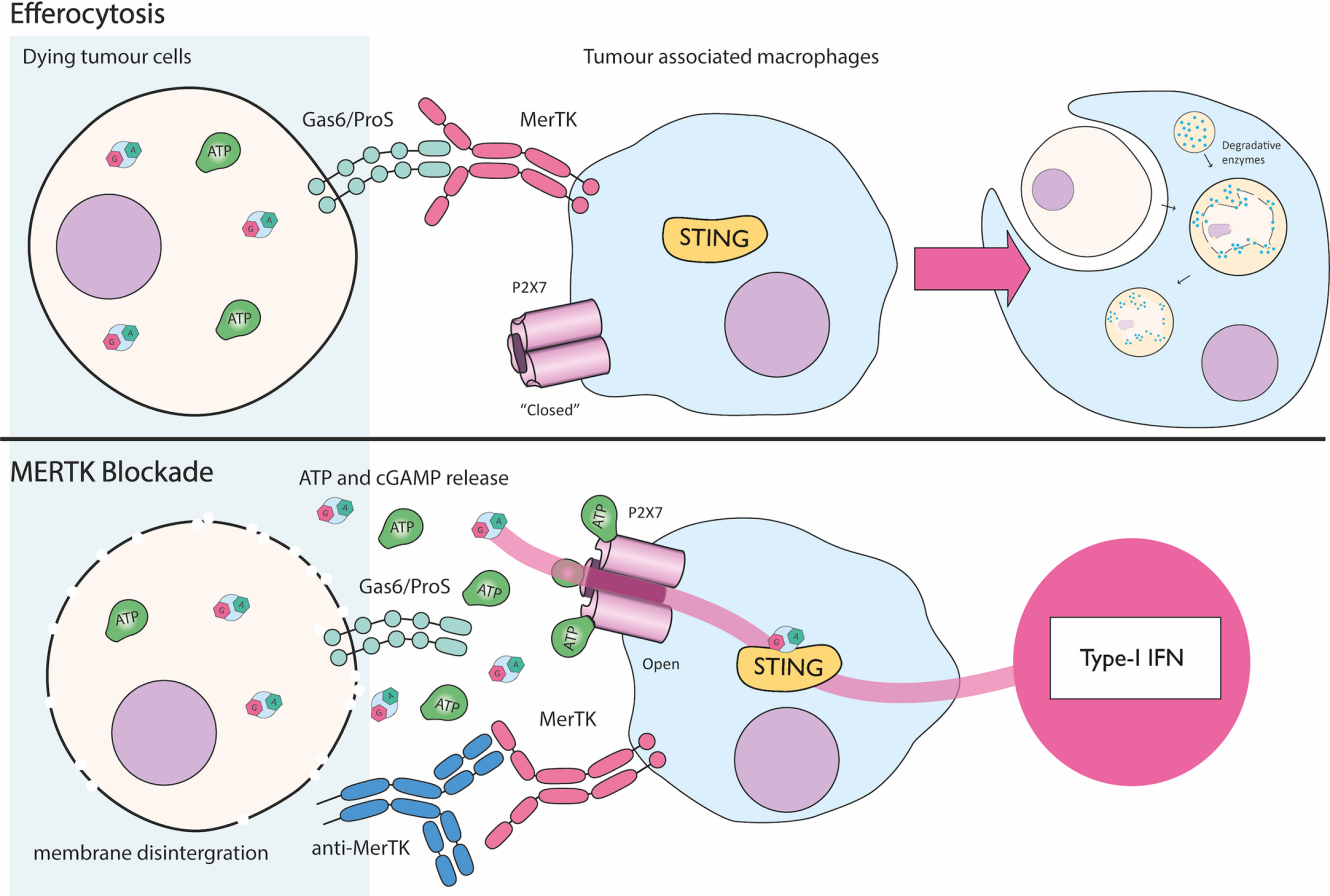
P2X7 mediated cGAMP uptake during MerTK blockade. Dying cancer cells are cleared by efferocytosis where the MerTK receptor present on tumor associated macrophages (TAMs) recognizes “eat me” signals Gas6/ProS. This leads to immunologically silent cell death by engulfment and digestion of the cellular corpse. Blockade of MerTK activity with an anti-MerTK antibody results in accumulation of dying cancer cells. Likely as a result of membrane disintegration, cGAMP and ATP are released into the tumor microenvironment. ATP activates P2X7 pore expressed by TAMs allowing cGAMP to passively move across the plasma membrane and drive type I IFN production through STING (1, 3–6, 24, 42–63).

P2X7 was first implicated in cGAMP transport by Zhou et al., in a model of efferocytosis of dying tumor cells (**Figure 5**) (20). Efferocytosis is usually mediated when a cell displays Gas6 or ProS on the cell surface. These “eat me” molecules trigger monocytically expressed MerTK to induce phagocytosis and immunologically silent cell death. The authors observed in two mouse models of cancer (MC38, colon adenocarcinoma; and E0771, triple negative breast cancer) that blocking MerTK mediated efferocytosis by tumor associated macrophages causes release of cGAMP from dying cells. They speculate membrane integrity eventually becomes disrupted in dead cells that are not cleared and hence cGAMP is released into the extracellular medium. *In vivo*, MerTK antagonism reduces tumor size over time and acts cooperatively with other immunotherapies (e.g. PD-1 blockade) (20). This effect requires the engrafted tumor to have active cGAS and the host to have STING suggesting a transfer of cGAMP from tumors to host cells. P2X7 was identified as the channel mediating exogenous cGAMP uptake into host tumor-associated macrophages, since P2X7 knockout prevented the ISG signature seen in the tumor microenvironment and stopped tumor regression in response to MerTK blockade. Additionally, in bone marrow derived macrophages, ATP strikingly enhances STING activation upon treatment with extracellular cGAMP. This effect is nullified by the P2X7 specific inhibitor A740003, indicating murine P2X7 likely imports cGAMP in this system (20). Whether human P2X7 also imports cGAMP has yet to be shown. An additional controversy relates to the general mechanism of P2X7 function as it is currently debated whether P2X7 itself forms a pore, or instead if it recruits a currently unknown pore forming protein (64).

#### 2.1.5 Volume regulated anion channels (LRRC8A:C/E)

Maintenance of cellular volume in response to extracellular osmotic change is critical to cellular homeostasis. Volume regulated anion channels (VRAC) are named after the observable efflux of Cl^-^ and organic solutes when cells are placed in a hypotonic (low salt) solution, which induce cell swelling (65, 66). The release of anions induces the osmotic efflux of water and so reduces/maintains cellular volume. Three decades after this electro-osmotic phenomenon was observed, the protein which forms VRAC channels was identified - LRRC8A (also known as SWELL1) (67, 68). Structurally, LRRC8A forms a hexameric channel into which the other LRRC8 members, LRRC8B-E, are stochastically incorporated. LRRC8A is the critical component and a heteromeric hexamer must be formed to efficiently mediate anion transport across membranes (69). All VRACs appear to convey small anions such as chloride, but hexamer composition determines the larger species transported. For instance, complexes containing LRRC8A:B mediate inorganic anion flux, whereas LRRC8A:C and LRRC8A:E also transport larger anionic osmolytes such as ATP, glutamate, and aspartate. Finally, LRRC8A:D has generally increased permeability for neutral and even positively charged osmolytes such as: taurine, lysine, serine, inositol, γ-aminobutyric acid (GABA) and the anticancer drug cisplatin (70–73).

A CRISPR screen that aimed to identify the transporter mediating background cGAMP uptake in SLC19A1 KO cells identified LRRC8A as a potential cGAMP importer in humans (18). Knockout of the VRAC subunits LRRC8A, LRRC8C and LRRC8E reduces STING activation in response to exogenous cGAMP. In HEK293 cells, hypotonic buffers enhanced STING activation in response to extracellular cGAMP in an LRRC8A:C/E dependent manner. Additionally, patch clamp analysis showed cGAMP was able to competitively inhibit the influx of Cl^-^ ions through VRAC, suggesting cGAMP permeates LRRC8A:C/E (18). Surprisingly, knockdown of LRRC8D increases STING activation in response to extracellular cGAMP, suggesting LRRC8D inhibits cGAMP transport through the LRRC8A:C/E complex (18). Mechanistically, LRRC8D likely competes with LRRC8C/LRRC8E for the available pool of LRRC8A by forming a pore with enhanced permeability for neutral or positively charged species, which blocks negatively charged cGAMP from moving through the channel (71, 73, 74). To find cells in which the LRRC8A:C/E channel imports cGAMP under physiological conditions, Lahey et al. looked for cell lines with high expression of LRRC8A and LRRC8C but low expression of the inhibitory subunit LRRC8D. Primary human umbilical vein endothelial cells (HUVECs) were found to fit this profile, leading initial reports to suggest that, in humans, LRRC8A:C/E mediated cGAMP import is limited to vascular endothelial cells (18). In addition to osmotic gradients, VRACs are also activated by sphingosine-1-phospahte (S1P) via the G protein coupled receptor S1PR1. S1P, like hypotonicity, was shown to enhance cGAMP transport through LRRC8A:C/E, increasing STING activation (18).

Despite efforts predominately focusing on import, VRACs have also been shown to act as cGAMP exporters. HEK293 cells overexpressing cGAS release cGAMP upon treatment with hypotonic buffers in an LRRC8A dependent manner (18). The disproportionate focus on import means LRRC8A:C/E mediated cGAMP export is relatively understudied. Future work will be needed to test if LRRC8A:C/E can export cGAMP when cGAS is expressed endogenously and the physiological relevance of this.

It has been suggested that cGAMP transport through LRRC8A:C/E only occurs under “artificial hypotonic conditions” and therefore may not be physiologically relevant (21). As osmotic pressure is relative, we believe the import or export of osmolytes could trigger LRRC8A:C/E activity under physiological conditions. Additionally, the secondary channel agonist S1P exists endogenously and therefore provides a mechanism for LRRC8A:C/E activation. A physiological role for VRAC mediated cGAMP transport is supported by studies in mice showing LRRC8A:C/E mediated cGAMP uptake. Unlike in humans, cGAMP import in mice is not limited to endothelial cells as murine fibroblasts, macrophages and T cells all import cGAMP through LRRC8A:C/E (**Figure 3**) (19, 75). Additionally, *in vivo* studies show LRRC8A:C/E mediated cGAMP import restricts HSV-1 infection and play a role in experimental autoimmune encephalitis (discussed in detail in sections II.1 and II.4) proving LRRC8A:C/E is physiologically relevant (19, 75). Since LRRC8A:C/E activity can be affected by osmotic disruption, studies looking to identify or characterize cGAMP channels should carefully consider if transport could occur indirectly through LRRC8A:C/E. Particularly as over expression or knockout of ion channels could lead to disrupted osmotic homeostasis, a potential trigger for LRRC8A:C/E mediated cGAMP transport.

#### 2.1.6 ABCC1, a cGAMP exporter

The ABC transporter family encompasses 49 members classified into 7 subfamilies (ABCA-G) (76). Each ABC transporter contains two cytosolic nucleotide bindings domains, which hydrolyze ATP to actively transport a broad range of substrates across the membrane. ABCC1 [also known as multidrug resistance protein 1 (MRP1)] contains 17 transmembrane domains forming a pore through which substrates are transported (25). ABCC1 exports a wide variety of endogenous substrates: Leukotriene C4, lipid species including S1P, glucuronides, glutathione conjugates and free glutathione [reviewed in (25)]. The physiological role of ABCC1 is ill defined. Some have suggested ABCC1 exports xenobiotics and toxic by-products of metabolism (77). This is in line with ABCC1’s polarized expression profile at barrier sites within the body such as choroid cells which maintain separation between the blood and cerebrospinal fluid; capillary endothelial cells forming the blood brain barrier; and syncytiotrophoblasts within the placenta, which separate mother from fetus (78–80).

ABCC1 was first implicated in cGAMP export by Maltbaek et al., who observed cGAMP export from cells after transfection with dsDNA and screened different classes of exporter to identify this transporter (81). ABCC1 has mainly been studied in the context of autoimmunity. TREX1 is a 3’ → 5’ exonuclease, which degrades any DNA that inadvertently reaches the cytosol under sterile conditions. Mutation of the *TREX1* gene leads to the chronic interferonopathy Aicardi-Goutières syndrome due to activation of cGAS (42, 82, 83). In accord, *Trex1* -/- mice have an average life span of ~110 days due to the associated severe inflammatory phenotype (21, 42). Maltbaek et al., show the type-I IFN signature and immunopathology seen in *Trex1* -/- mice is exacerbated when *ABCC1* is simultaneously knocked out. *In vitro*, ABCC1 was shown to export cGAMP in an ATP dependent manner. ABCC1 mediated cGAMP export negatively regulates IFN induction by preventing cGAMP binding to STING (21). Therefore, ABCC1 is the only cGAMP transporter shown to reduce IFN induction, since all the cGAMP importers are considered to function in a proinflammatory manner. Export of cGAMP leading to reduced STING activation aligns with the only known mechanism of cGAMP degradation - ENPP1 - being extracellular. Whether ABCC1 can also function to enhance cGAMP driven responses in settings of microbial invasion or cancer remains to be seen. Additionally, if cGAMP export through LRRC8A:C/E also acts to alleviate STING driven inflammation has not been investigated. A known substrate of ABCC1, S1P, is a known agonist of LRRC8A:C/E. Therefore, whether ABCC1 acts cooperatively with LRRC8A:C/E is an interesting possibility (18, 84). Another ABC family member - ABCG2 - has been proposed as a cGAMP exporter and chemical inhibition was previously shown to prevent cGAMP export (21). As ABCC1 and ABCG2 are the only ABC transporters known to export S1P, this further suggests a link between ABC and LRRC8A:C/E mediated cGAMP transport (84). However, ABCC1 has been shown to export cGAMP from “inside out” vesicles derived from insect cell lines devoid of LRRC8A:C/E, indicating that ABCC1 is likely a cGAMP conduit in its own right and not indirectly acting by opening LRRC8A:C/E via S1P (21).

#### 2.1.7 LL-37

Antimicrobial defense peptides (AMP) are small cationic amphipathic molecules found across all kingdoms of life. They are split broadly into two classes: cathelicidins and defensins (85). The AMPs found in multicellular organisms resemble the offensive peptides released by prokaryotes to kill competing microorganisms and are likely one of the earliest forms of immune defense. LL-37 is the only human encoded member of the cathelicidin family and is only 4.4 kDa in size (86). It exists as a proprotein, hCAP-18, and is expressed by front line immune cells such as neutrophils, macrophages, dendritic cells, natural killer cells, mast cells and epithelial cells (87). LL-37 is released into the site of inflammation by exocytosis and is cleaved by serine proteases into its active form (88, 89). Within bacterial cell membranes, LL-37 forms a tetrameric pore leading to bacterial cell lysis (90–92). Host cell membranes are protected from pore formation due to elevated cholesterol levels, but high concentrations of LL-37 can still lead to membrane permeabilization (93). Alongside direct antibacterial activity LL-37 also acts as: a chemoattractant for macrophages, dendritic cells and neutrophils; a catalyst of NLRP3 inflammasome activation; a trigger for degranulation of mast cells; and is a cytokine signaling through EGFR, FPRL-1 and even P2X7 (87, 94, 95). Additionally, LL-37 can form a complex with extracellular dsDNA able to activate endosomal TLR9 in pDCs and drive STING activation through cytoplasmic DNA sensors in monocytes (96, 97). The LL-37-dsDNA complex reaches endosomes through lipid raft and proteoglycan dependent endocytosis, but how it crosses the endosomal membrane to reach cytosolic DNA sensors is unknown (98). However, it has been suggested that the cationic amphipathic complex might be able to passively diffuse through the phospholipid bilayer (87, 96).

A recent study has shown that LL-37 also transports cGAMP across the cell membrane, since *in vitro* treatment of cells with extracellular cGAMP and LL-37 enhances STING activation (22). *In vivo*, prophylactic treatment of mice with LL-37 and extracellular cGAMP has been shown to restrict HSV-1 replication and increase IFN driven responses (22). However, the mechanism by which exogenous LL-37 delivers cGAMP to the cytosol is unclear. cGAMP, like dsDNA, forms a complex with LL-37 potentially able to cross cell membranes (22). Equally, tetrameric pore formation could allow cGAMP to access the cytosol through nonspecific membrane disruption. Cells treated with LL-37 do not show increased permeability to propidium iodide (PI) suggesting that, in this context, cellular membranes are not disrupted (22). However, as PI is a positively charged molecule, it may be unable to move through the positively charged LL-37 pore (92, 99). Therefore, whether cGAMP passes through the LL-37 pore or moves through the membrane as a cGAMP-LL-37 complex remains uncertain. Future work assessing if endogenously expressed LL-37 enhances cGAMP mediated responses will be crucial to prove a physiological role for LL-37 during cGAS-STING signaling. Interestingly, dsDNA-LL-37 complexes are resistant to nuclease degradation (98). Therefore, it remains to be determined if LL-37 protects cGAMP from ENPP1 mediated hydrolysis.

### 2.2 Topological cGAMP transport

cGAMP can also be transported between cells via topologically contained mechanisms, meaning that it does not transit through the extracellular space and is therefore protected from ENPP1 hydrolysis. These mechanisms include the transfer to juxtaposed cells via GAP junctions and to neighboring or distant cells via incorporation into viral particles.

#### 2.2.1 Local signaling through GAP-junctions

Gap junctions are intercellular channels which effectively link the cytoplasm of one cell to its neighbor. Their function is broad, playing a role in developmental signaling, electrical potentiation, cellular proliferation and differentiation (100). They are formed by connexins of which there are 21 known in humans. These form hexamers known as “hemichannels” (101). A hemichannel on one cell links to another on an adjacent cell forming a gap junction. These assemble in clusters to form hundreds of intercellular channels, allowing for the passage of small and hydrophilic species (<~1.2 kDa), including signaling molecules, between cells (e.g. ATP, cAMP, IP_3_, glucose, glutathione, glutamate, Ca^2+^, K^+^ and Na^+^) (102). This constant exchange of metabolites and signaling molecules is called gap junction intercellular communication (GJIC) and allows a tissue to act as a collective. GJIC is a basic mechanism of tissue homeostasis and explains GAP junction’s wide ranging physiological role.

It was known half a decade before the discovery of cGAS that GAP junctions facilitate IFN-β production in response to dsDNA. This was shown in HEK293 cells expressing GFP under the control of an IRF3 promoter. Upon dsDNA transfection, a striking visual pattern is seen where clusters of cells become GFP positive, suggesting stimulated cells might be instigating innate immune activation in their physically associated neighbors. This effect is lost on siRNA depletion of GAP junction components (103). Shortly after cGAMP discovery, it was identified as the messenger responsible, as knocking out the only two connexins present in HEK293 cells (CX43 and CX45) prevented cGAMP transfer. Upon cellular reconstitution with other known connexins (CX26, CX31, CX32, CX40, CX52a and CX50) all but CX50 rescued cGAMP transfer. This is in line with the notion that gap junctions form pores with limited cargo specificity so redundantly facilitate cGAMP transfer between cells (8). Remarkably, gap junctions can also transfer cGAMP between non-static cells. In coculture experiments, HEK293 cells could transfer cGAMP to primary human monocytes, a process dependent on expression of CX43 and CX45 (43). Additionally, *in vivo*, cGAMP coated in pulmonary surfactant mimetic liposomes (PS-cGAMP) can be taken up by alveolar macrophages and the cGAMP transferred to alveolar epithelial cells through GAP junctions (104). This passing of cyclic dinucleotides by non-static cells complicates interpretation of *in vivo* models since the cells reacting to cGAMP may not be directly importing the cGAMP from the extracellular space.

Hemichannels at the cell surface have also been shown to have central roles in autocrine and paracrine signaling, allowing exchange of small molecules between the cytoplasm and surrounding tissue fluid. This facilitates movement of small metabolites (e.g. ATP, glutamate, NAD^+^, IP_3_, prostaglandin E_2_, and cAMP) across the cell membrane (105–107). The cyclic dinucleotide cAMP has a similar size and structure to cGAMP (~0.7 KDa) (106). However, as of yet, the role hemichannels play in cGAMP export/import has not been investigated.

#### 2.2.2 Long-range signaling: cGAMP incorporation into viral particles

In virus infected cells, viral particles assemble on cellular membranes. They are made up of viral proteins that encapsidate the viral genome to transport it into the next target cell. Extensive proteomic and lipidomic analyses of viral particles from different viruses have shown that they can also incorporate cellular proteins, lipids or RNAs (108). These host components are thereby transmitted to the next infected cell where they can affect both viral replication and cellular responses. cGAMP was recently shown to be among the small molecules known to be transported in this manner. The authors were initially investigating if cGAS, like other cellular proteins, could be packaged into lentiviral virions (10). They and others then observed that viruses [vaccinia virus (VACV), murine cytomegalovirus (MCMV) and human immunodeficiency virus 1 (HIV-1)] produced in cGAS expressing cells triggered STING activation upon viral entry into new cells (9, 10). Surprisingly, the IFN response in target cells was independent of dsDNA production in the target cells, ruling out cGAS packaging in the viral particles. Instead, they found that cGAMP was incorporated within virions and was being transferred between cells. These observations are remarkable when considering the very small volume of a viral particle relative to a cell. Therefore, it is astonishing that enough cGAMP can be delivered to activate STING.

Similar to virus particles, extracellular vesicles (EVs), including exosomes and microvesicles, are vesicular structures produced by most cells that are involved in intercellular communications (109). They can transport cellular proteins, lipids or RNA to neighboring cells but also very distant cells in other organs. They can be secreted by most cells and are therefore present in all organs and body fluids. They have known roles in key biological processes such as vascular biology, pregnancy, embryonic development, tissue repair, bone calcification, liver homeostasis, functions of the nervous system and immune responses (109). Changes in EVs composition are associated with various pathologies including cancer, which lead to their use as biomarkers. Moreover, they also play a direct role in pathogenesis in a variety of diseases depending on the cargo they transfer to neighboring cells. For example, EVs produced by tumor cells function to sustain the proliferative signaling, promote invasion and metastasis, induce angiogenesis and reduce the activity of immune cytotoxic cells in the tumor microenvironment (110). Interestingly, EVs have been shown to carry important soluble mediators such as cytokines. Therefore, as EVs are similar to virus particles it is interesting to speculate that they could also incorporate cGAMP. One study generated cGAMP-containing exosomes by incubating HEK293T cells-derived purified exosomes with free cGAMP for 16h (111). The cGAMP signal in the loaded exosomes was resistant to treatment with the cGAMP hydrolase snake venom phosphodiesterase (SVPDE), suggesting that cGAMP was indeed inside the exosomes. In this context, incorporation of cGAMP was independent of the known importer SLC19A1 but the authors did not exclude a contribution from other conduits. The incorporation of cGAMP into EVs released by cells where cGAS is activated and produces cGAMP was suggested by one study (9). Here, the authors found cGAMP in the EVs fraction of supernatant from DNA-transfected cells that were not producing viral particles. However, unlike supernatants containing viral particles, these cGAMP-EVs containing supernatants were not able to induce IFN responses in the PMA-treated THP-1 model of macrophages or in HEK293FT cells. Therefore, whether cGAMP-containing EVs can transfer cGAMP to new target cells requires further investigation and this represents an exciting area for future study.

## 3 cGAMP intercellular signaling in disease and potential for therapies

A role for the cGAS-STING pathway has been described in inflammation and anti-microbial response in many diseases [reviewed in (112)] and others are likely still unknown. In instances where cGAS is activated and produces cGAMP, cGAMP transport will likely participate in tissue inflammation via the mechanisms described in this review. However, the description of cGAMP transport between cells is recent and a role for cGAMP transport, topological or extracellular, in diseases remains largely unknown. This is important because, the revelation that cGAMP is transported between cells has particular therapeutic potential outlined in this section.

### 3.1 Cancer

#### 3.1.1 cGAMP transport and immune responses to cancer

Many cancers have unstable nuclei due to incomplete chromosomal separation during mitosis (44). This leads to the formation of micronuclei, enclosing DNA within “leaky” membranes accessible by cGAS (113, 114), usually resulting in immune responses acting to suppress tumor growth. Indeed, cGAMP transport between cancer cells and the host cells induces an immune response able to reject the tumor in mice. This has been shown using different models of tumors where the cancer cells need to express cGAS and the host cells only need to express STING to initiate responses leading to tumor rejection (20, 26, 115, 116). However, cancer cells also express ENPP1 which promotes metastasis by hydrolyzing extracellular cGAMP in the tumor microenvironment (117). In this model, loss of ENPP1 in the tumor inhibited metastasis and restored immune infiltration and sensitivity to treatment with immune checkpoint inhibitors, which required cGAS in the tumor cells and STING in the host cells. cGAMP transport is therefore a key signal for the recruitment of immune cells to the tumor. Mechanistically, cGAMP was shown to be transferred between cancer and host cells via gap junctions or as extracellular cGAMP via currently unidentified transporters. Characterizing these transporters could guide the design of targeted cGAMP delivery to promote anti-tumoral immune responses. cGAMP transport via gap junctions was shown to have opposite effects in different models. Indeed, in a mouse model of colon cancer, tumor cells transferred cGAMP to tumor-associated dendritic cells and macrophages via gap junctions thereby inducing an anti-tumor CD8 T cells response (116). However, transfer of cGAMP via gap junctions was also shown to have a pro-tumoral effect in a model of brain-invading lung and breast cancer (45). In this model, the tumor cells transferred cGAMP via gap junctions (CX43) to astrocytes which led to astrocyte-driven production of IFN-α and TNF-α driving the growth and chemoresistance of the invading tumor. In line with these findings, the clinically approved inhibitors of GAP junctions (Meclofenamate and Tonastat) were able to significantly reduce invasive brain tumor growth in mice, thereby proving antagonist of cGAMP transport have clinical relevance for cancer treatment (45). The use of immunotherapies targeting cGAMP transport therefore needs to be carefully evaluated in different models.

#### 3.1.2 ENPP1 inhibitors

Many tumors secrete cGAMP in the tumor microenvironment but, interestingly, cGAMP efflux from cancer cell lines can only be observed upon chemical inhibition or knockdown of ENPP1. In accord, *Enpp1*-/- mice unable to degrade cGAMP are resistant to tumor development in multiple models of cancer (26). Tumor resistance is also observed in mice treated with ENPP1 inhibitors, an effect enhanced by simultaneous radiotherapy, a treatment known to increase erroneous chromosomal segregation and so augmenting detection of self-DNA by cGAS (26, 44, 113, 114, 118, 119). Overall, ENPP1 potently suppresses the proinflammatory effects of extracellular cGAMP within the tumor microenvironment. Therefore, ENPP1 inhibition represents an exceptional therapeutic strategy as endogenous cGAMP production would then be localized to the tumor microenvironment. Systemic treatment with ENPP1 inhibitors sidesteps clinical concern regarding tumor disruption during intratumoral injection of therapeutic CDNs, which may physically dislodge cancerous cells encouraging metastasis. cGAMP and ATP degradation mediated by ENPP1 also contributes to the extracellular pool of adenosine, an immunosuppressant detected by the adenosine receptor. Extracellular adenosine produced by ENPP1 promotes both tumor growth and metastasis (117). Therefore, ENPP1 inhibitors would act to simultaneously augment STING activation and prevent adenosine’s anti-inflammatory properties. Additionally, since ENPP1 hydrolyses both ATP and cGAMP, ENPP1 inhibition may allow for accumulation of extracellular ATP and the activation of P2X7 pores. This raises the exciting possibility that ENPP1 inhibitors could act synergistically with MerTK blockade to clear cancer (see section I.1.4). The tremendous therapeutic promise of ENPP1 inhibitors is well illustrated by swift financial interest from pharmaceutical investors. Professor Lingyin Li, the true pioneer of extracellular cGAMP responses, recently raised a $11.3 million in funding to create Angarus, a company focused on the development of ENPP1 inhibitors, the first of which - AG-3132 - is about to enter phase 1 clinical trials (120).

#### 3.1.3 Direct injection of CDNs into tumors

As an alternative strategy, direct injection of cGAMP in the tumors has been studied, especially in the context of cancer vaccines (see section II.2) but it is limited by the presence of ENPP1. Another way of triggering STING responses in the tumor-microenvironment while by-passing ENPP1 degradation is to use the recently developed modified CDNs that are resistant to ENPP1 degradation. Indeed, efforts have been made to produce non hydrolysable cGAMP analogues by replacement of phosphodiester linkages with phosphothioate linkages (e.g. 2’3’-cG^S^AMP, 2’3’-cGA^S^MP, 2’3’-cG^S^A^S^MP). This proved efficacious as phosphothioate analogues are 40 times more resistant to ENPP1 hydrolysis and induce more potent STING activation in cell culture (27). Additionally, 3’3’ linked species (e.g. 3’3’-cGAMP) produced by bacteria as secondary messengers have also shown resistance to ENPP1 mediated hydrolysis (27). This is the strategy adopted by companies developing CDN based immunotherapeutics, which are currently in clinical trials. Notably, the CDNs 2’3’CDA^S^ (also known as ADU-S100) and MK-1454 are being tested for activity against advanced/metastatic solid tumors and lymphomas (trial IDs -NCT03172936 and NCT03010176) (12, 121). As these CDNs show different specificities for the cGAMP conduits, knowing which cell types express the different cGAMP conduits could allow for cell targeted CDN treatments based on CDN channel expression profiles (12). This is of particular importance for the development of these CDN-based therapies as CDN mediated STING activation can inhibit the proliferation of T cells required for effective tumor rejection. For example, 3’3’-cGAMP appears to be taken up better by dendritic cells and macrophages but not T cells, thereby stimulating antigen presentation and possibly providing the best therapeutic outcome. Collectively, injection of CDNs into tumors has shown therapeutic promise but its effects are largely hindered by poor induction of systemic T cell responses needed for cancer clearance.

### 3.2 Applications for vaccine development

#### 3.2.1 Free cGAMP as a novel adjuvant

Many studies have tested the use of cGAMP as an adjuvant for anti-cancer or anti-microbial vaccines. These have been recently extensively reviewed in Van Herck, Feng (122) and Garland, Sheehy (123). In this review, we will mostly focus on the literature showing the advantage of exploiting cGAMP transport mechanisms for innovative vaccine design. When free cGAMP is co-administered with a vaccine, it enhances immune responses to the antigens and therefore is a potent adjuvant. This was demonstrated using different routes of administration (intra-muscular, intra-dermal, intra-veinous or intra-tumoral) and in very different disease context such as anti-tumor therapies and prophylactic anti-viral vaccines. All vaccine strategies that use free cGAMP as an adjuvant rely on cGAMP importers on the target cells in order for cGAMP to have an effect. The cell types expression profile of the channels might therefore determine the specificity of cell types targeted by the injected free cGAMP. However, the specific transporters required for the adjuvant effects and their cell type specificity have not been investigated. Some studies using free cGAMP as an adjuvant in the lungs found that it can induce unwanted responses such as asthma or increased viremia following challenge with porcine reproductive and respiratory syndrome virus (124, 125). Other studies using similar strategies did not find these adverse effects suggesting that these could be due to the specificities of the systems used (126–129). Moreover, in all these conditions, cGAMP needs to be used at high concentrations. This is due to a short half-life of this molecule *in vivo* depending on the route of injection (130). However, recent studies suggest that high doses of CDNs inhibit proliferation of T lymphocytes that are required for an efficient adaptive immune response (in this case, an anti-tumor response) (75). Collectively, these studies suggest that injecting free cGAMP lacks a certain specificity and could lead to detrimental outcomes. ENPP1 is likely responsible for the short life of free cGAMP in organs. Several solutions have been proposed to this problem (1): developing cGAMP analogs that are resistant to ENPP1 hydrolysis but would still use cGAMP transporters and activate STING (see section II.1.3) and (2) incorporating cGAMP in various delivery systems to protect it from ENPP1 hydrolysis.

#### 3.2.2 Enhanced efficiency of cGAMP as an adjuvant in various delivery systems

Several studies formally comparing free cGAMP and cGAMP administered within nanoparticles show that encapsulating cGAMP increases its adjuvant potential and allows to reduce the dose of vaccine needed to achieve the same protection (104, 131, 132). Knowing that cGAMP can be incorporated into virus particles, our laboratory used viral vaccine vectors loaded with cGAMP in a mice model of vaccination and were able to show that incorporation of cGAMP in the viral particles can increase the systemic immune response to the target antigens compared to Empty-VLPs adjuvanted with free cGAMP (133). Associating the adjuvant cGAMP with viral proteins in the vector was therefore a successful antiviral vaccine strategy. A recent study associated cGAMP with mRNAs from Influenza in lipid nanoparticles and showed an increased innate and adaptive immune response to the vaccine in the presence of cGAMP (132). Similarly, pulmonary surfactant mimetic microsomes containing cGAMP used as an adjuvant alongside influenza vaccination and administered intra-nasally greatly enhanced humoral and CD8 T cell responses in mice (104). Interestingly, in this study, the cGAMP-containing microsomes were taken up by alveolar macrophages, and cGAMP was then transferred to neighboring alveolar epithelial cells via gap junctions and activated STING in both cell types. In contrast to the currently available flu vaccines, these last two vaccines provided cross protection against distant and heterosubtypic flu strains, a remarkable and long sought-after accomplishment. Packaging cGAMP in a delivery system also represents a promising strategy to target cGAMP to tumor-infiltrating dendritic cells. Indeed, as demonstrated in a recent study, intratumoral injection of cGAMP-containing virus-like particles (VLP) specifically activated STING in the dendritic cells (134). This led to systemic anti-tumor T cell responses able to synergize with immune checkpoint blockade or tumor Treg depletion to clear the tumor in multiple murine models. Direct comparison of cGAMP-VLP with the synthetic CDN ADU-S100 showed that this compound induced necrosis and cleared the tumors but failed to induce a systemic anti-tumor T cell response. The authors suggest that cGAMP loading into VLPs allows to reduce the dose of cGAMP injected which likely contributes to the lower tissue necrosis and to target cGAMP to dendritic cells.

Importantly, several systems using cGAMP as an adjuvant have shown enhanced protection in two populations that usually respond poorly to vaccines and are therefore difficult to protect against infections: early-life and aged mice (135–137). Collectively, these studies clearly illustrate the immense therapeutic potential of cGAMP containing vaccines.

### 3.3 cGAMP transport enhances anti-microbial responses

The relationship between bacterial infections and the cGAS/STING pathway are very complex (138). Depending on the bacteria, activation of the cGAS/STING pathway can either help fight the infection or increase susceptibility of the infected host. cGAS can be activated by bacterial DNA or via release of mitochondrial DNA following stress. Moreover, bacteria produce their own CDNs that are injected into the infected cell cytosol by bacterial secretion systems or following partial lysis in phagosomes (138). These bacterial CDNs are then directly sensed by STING in a cGAS-independent manner. This demonstrates that STING can act as a PRR in its own right. *In vitro*, CDNs can pass through the cellular membrane via the cGAMP conduits described in this review as detailed in **Figure 3** but whether this occurs during a bacterial infection has not been studied. A role for 2’3’-cGAMP transfer in bacterial infection has also not been fully explored. One study showed that transfer of the cGAS-STING signal between cells is responsible for an IFN response in *Chlamydia* infection (139). Indeed, infecting cells depleted for cGAS or for STING does not lead to any IFN production, ruling out a role for an activation of STING by bacterial cyclic dinucleotides. However, when infecting a mix of cells depleted for cGAS (cGAS-/STING+) and cells depleted for STING (cGAS+/STING-), IFN is produced, probably via a transfer of cGAMP from the cGAS competent cells to the STING competent cells. Therefore, cGAMP produced during *Chlamydia* infection can be transferred between cells but the mechanism involved and the anti-microbial effect of this transfer were not investigated (139). Transfer of CDNs, including 2’3’-cGAMP, between cells and their effect on bacterial replication and cellular responses need to be further investigated to determine if they represent promising therapeutic targets.

As previously mentioned (see section I.2.2), the transport of cGAMP via viral particles was described for a number of viruses (e.g. VACV, MCMV and HIV-1) when they are produced in cGAS-competent cells (9, 10). Other enveloped viruses that activate the cGAS-STING pathway might therefore transfer cGAMP via this mechanism. Monocytes pre-treated with virus particles containing cGAMP were resistant to further infection with another virus, which demonstrates that cGAMP-loaded viral particles have the potential to induce an antiviral state in the recipient cells (9). However, whether cGAMP is really incorporated in viral particles *in vivo* and if this has an effect on viral replication is not known. *In vitro*, cells infected with vaccinia virus transfer cGAMP to neighboring cells via gap junctions which induces an antiviral state in bystander cells and limits viral spread (8). Further studies are required to investigate the significance of this mechanism *in vivo*. Extracellular cGAMP also plays a complex role in viral infections. It restricts replication of the DNA virus Herpes Simplex Virus 1 (HSV-1), as mice expressing the mutant ENPP1 (H362A) unable to degrade cGAMP have elevated extracellular cGAMP levels and are resistant to HSV-1 infection in a STING dependent manner (140). Similarly, mice lacking the anion channel LRRC8E, are more susceptible to HSV-1 infection (19). However, in the context of infection with the RNA virus Influenza A Virus (IAV), mice lacking the anion channel LRRC8C that drives cGAMP import into T cells, showed a lower level of viral replication associated with a higher IAV-specific antibody response (75). As LRRC8C drives import of cGAMP into T cells, resulting in inhibition of T cell function, this suggests that blocking LRRC8C might be beneficial in RNA virus infection. These two examples also show that, in mice, extracellular cGAMP transport through VRACs can have opposite effects on the antiviral immune response that might depend on the source of cGAS activation and on the cell types involved. Whether this is also the case in humans remains to be explored. More evidence for a direct role of inter-cellular cGAMP signaling in the context of other virus infections is also still required.

The arms race between immune responses and pathogens results in the development of microbial strategies to evade immune responses that impact their replication or spread. The existence of such a microbial strategy represents a clue pointing toward the importance of an immune mechanism in antimicrobial responses. Targeting cGAMP specifically might be an efficient strategy for viruses to both inhibit IFN production by the infected cell and limit the spread of the IFN response to neighboring cells. A few viruses have now been shown to directly degrade cGAMP to avoid antiviral signaling in the infected cell but also probably to limit cGAMP transfer to neighboring cells using the various mechanisms described in this review. Poxviruses such as vaccinia virus express an enzyme called poxvirus immune nuclease (poxin) (141). This enzyme degrades cGAMP *in vitro*, decreases cGAS-STING signaling in cells and is essential for vaccinia virus replication and spread *in vivo*. Similarly, the African swine fever virus encodes two proteins (C129R and EP364R) that degrade cGAMP in infected cells (142). Whether these virally encoded cGAMP degrading enzymes act extracellularly is unknown. Moreover, whether viruses have also evolved ways to inhibit the cGAMP channel activity remains to be seen but if cGAMP channels function within antiviral immunity, antagonists will likely exist.

### 3.4 Auto-immune, inflammatory and other diseases

Although operational DNA sensing is required for protection from microbial threats, overzealous responses drive chronic proinflammatory conditions. The prototypical examples of STING mediated autoimmune disease are the debilitating interferonopathies: Aicardi-Goutières syndrome (AGS), STING associated vasculopathy with onset in infancy (SAVI) and COPA Syndrome (1–3). These are all caused by chronic activation of the cGAS-STING pathway. AGS is caused by mutations in genes (*SAMHD1, ADAR1, TREX1, IFIH1, RNASEH2A, RNASEH2B, RNASEH2C, LSM11*, and *RNU7-1*) many of which lead to accumulation of DNA in the cytosol that activates cGAS signaling (143). SAVI is caused by gain of function mutations in the *STING* gene. COPA syndrome is a disease where mutations in COPA, a component of COP-1 vesicles, result in STING being unable to traffic back from ERGIC and so chronically signals (1). However, in both SAVI and COPA syndromes, if cGAMP is required for sustained activation of STING is still unknown. Moreover, in a wide range of more prevalent inflammatory and autoimmune disorders, cGAS-STING signaling is thought to contribute towards pathology summarized in **Figure 1A** (4–6). The role of cGAMP transport in most of these diseases has not yet been investigated.

In alcohol-related liver disease (ALD), the cGAS/STING pathway drives inflammation and ensuing liver injury (144). Mechanistically, cGAS is activated in hepatocytes and the inflammation signal is propagated by transfer of cGAMP to neighboring cells via gap junctions. In mice, small-molecule inhibition of the hepatic gap junction CX32 decreased hepatocyte injury, inflammation and oxidative stress showing that targeting gap junctions could be used as a therapy to treat ALD.

cGAMP conduits can also show anti-inflammatory effects in various models. In a *Trex1 -/-* model of AGS, knockout of the transporter ABCC1 enhances pathology as cGAMP is retained within cells enhancing STING activation (21). This demonstrates that ABCC1 export of cGAMP protects from pathogenesis in this model. Moreover, in a model of autoimmune encephalitis, the anion channel LRRC8A:C also limits pathogenesis. Indeed, *Lrrc8c* -/- mice showed normal T cell development but developed hyperactive T cell responses in experimental autoimmune encephalomyelitis (EAE), a murine model of multiple sclerosis (75). Therefore, extracellular cGAMP uptake through LRRC8A:C limits T cell proliferation and survival in mice which is essential to suppress autoreactive T cells in auto-immune responses. In these examples, transfer of extracellular cGAMP via the conduits is actually beneficial to the host by reducing inflammatory and auto-immune responses. Therefore, the role of cGAMP transfer in inflammatory and auto-immune diseases is likely to be complex and represents a significant knowledge gap within the field that needs to be addressed. This is of particular importance as clinically approved inhibitors of cGAMP conduits already exist, making it possible to rapidly provide the rationale to broadly inhibit cGAMP transfer (**Figure 3**) in order to alleviate pathology of the many cGAS-STING associated diseases (**Figure 1A**).

## 4 Considerations for future research

Finally, we would like to highlight some considerations and interesting questions that are currently unanswered within the field.

**The differences in cGAMP transport between humans and mice**. *In vivo* studies on cGAMP conduit function are complicated by the vast differences seen in cGAMP transport between humans and mice. For example, murine SLC19A1 is not a cGAMP transporter, LRRC8A:C/E mediated transport appears to be limited to endothelial cells in humans but not mice, and murine SLC46A2 appears to transport cGAMP but does not function in murine monocytes and macrophages like human SLC46A2 (12, 13, 17–19). It is very important that future studies consider these differences as conclusions drawn solely from mice will likely be misleading. Many studies looking at cGAMP import have focused on the cell types and CDN species that move through the conduits *ex vivo*. Therefore, a shift in focus towards research on cGAMP conduits at the patient level could be crucial to understanding their physiological relevance. We suggest that the study of polymorphisms could allow conduit function to be correlated to cases of cancer or microbial disease in patient cohorts.

**Is cGAMP export proinflammatory?** The notion that cGAMP export by ABCC1 acts to dampen STING activation, as proposed by Maltbaek and co-workers, is in line with the only known degradative mechanism of cGAMP, ENPP1, being extracellular (21, 27). This also creates a contradiction as the proinflammatory cGAMP importers only have access to cGAMP if it has been delivered to the extracellular environment. Thus, depending on context, it is likely ABCC1 could act in both a proinflammatory and anti-inflammatory manner. Yet, a proinflammatory role for ABCC1 remains to be proven. It also remains to be seen if, like ABBC1, the only other known cGAMP exporter, LRRC8A:C/E, can act to suppress STING signaling, as it has only been shown to act in a proinflammatory manner (18, 19).

**Are other immunological pores cGAMP transporters?** P2X7 is thought to form a non-specific pore through which cGAMP enters monocytes (20). Interestingly other non-specific pores and proteins that rupture the cells membrane are well known and function within the immune response, notably: MLKL, perforin, granzyme B and ninjurin 1 (145–147). Cellular permeabilization would also likely lead to cGAMP export but it remains to be seen if these proteins function to enhance cGAS-STING signaling.

**Can 2’-5’ oligoadenylate be transported between cells?** The only cGAS homolog in humans is oligoadenylate synthase (OAS), which senses viral dsRNA and produces 2’5’-linked oligoadenylate (2-5A). 2-5A activates RNase-L to indiscriminately cleave RNA (148). This acts to restrict viral replication but whether 2-5A, like cGAMP, can be transported through conduits, within viral particles, or even spread between cells is an exciting possibility that has not yet been investigated.

**Could other membrane enclosed compartments transport cGAMP?** Aside from viral particles, other membrane enclosed compartments are known to traffic between cells and so potentially transport cGAMP; notably, exosomes which are known to signal between cells by ferrying metabolites and RNA species. Whether exosomes and microvesicles also function to transport cGAMP remains to be seen. Additionally, endogenous retroviruses that encode Gag homologs have recently been reported to form virus-like particles and traffic between cells, most notably Arc, HERVK and PEG10 (149–151). If these, like infectious lentiviruses, are also able to package cGAMP has not been investigated.

**Could viroporin channels antagonize cGAMP packaging into virions?** cGAMP is packaged into viral particles leading to IFN production upon infection of a new cell (9, 10). Intriguingly, some viruses (e.g. HIV-1, IAV and SARS-CoV2) encode channels termed viroporins that are incorporated into their envelope (152). It is interesting to speculate whether viroporins or host channels incorporated into the viral envelope could act to export cGAMP from virions and evade the antiviral effects of cGAMP packaging.

## 5 Conclusion

It is now clear cGAMP acts beyond the cell in which it is produced, acting as a travelling messenger via a variety of pathways (topological and extracellular) to drive type-I IFN production in the new cell. These new mechanisms which spread cGAMP broaden the impact of cytosolic DNA sensing by cGAS: it can now activate STING, IRF3 and NFkB in neighboring as well as distant cells. As these pathways have been discover over the last decade, still a lot of open questions remain to be addressed including the precise mechanisms involved, the existence of other similar proteins (enzymes like ENPP1 or transporters), and in particular their roles in the diseases where the cGAS-STING pathway is activated. This is a fast-evolving field that will undoubtedly yield exciting breakthroughs in the coming years with very interesting therapeutic applications for varied diseases.

## Author contributions

HB and LC designed the study, wrote and revised the manuscript. HB produced all figures. LC initiated, supervised and supported the study. All authors approved the submitted version.

## References

[1] Deng Z Chong Z Law CS Mukai K Ho FO Martinu T. A defect in copi-mediated transport of sting causes immune dysregulation in copa syndrome. bioRxiv (2020) 217(11):e20201045. doi: 10.1101/2020.05.20.106500 PMC7596814 32725126

[2] Ahn J Ruiz P Barber GN. Intrinsic self-DNA triggers inflammatory disease dependent on sting. J Immunol (2014) 193(9):4634–42. doi: 10.4049/jimmunol.1401337 PMC5003413 25261479

[3] Liu Y Jesus AA Marrero B Yang D Ramsey SE Sanchez GAM. Activated sting in a vascular and pulmonary syndrome. N Engl J Med (2014) 371(6):507–18. doi: 10.1056/NEJMoa1312625 PMC4174543 25029335

[4] Sliter DA Martinez J Hao L Chen X Sun N Fischer TD. Parkin and Pink1 mitigate sting-induced inflammation. Nature (2018) 561(7722):258–62. doi: 10.1038/s41586-018-0448-9 PMC7362342 30135585

[5] An J Durcan L Karr RM Briggs TA Rice GI Teal TH. Expression of cyclic gmp-amp synthase in patients with systemic lupus erythematosus. Arthritis Rheumatol (2017) 69(4):800–7. doi: 10.1002/art.40002 27863149

[6] Ahn J Gutman D Saijo S Barber GN. Sting manifests self DNA-dependent inflammatory disease. Proc Natl Acad Sci USA (2012) 109(47):19386–91. doi: 10.1073/pnas.1215006109 PMC3511090 23132945

[7] Ablasser A Chen ZJ. Cgas in action: expanding roles in immunity and inflammation. Science (2019) 363(6431):eaat8657. doi: 10.1126/science.aat8657 30846571

[8] Ablasser A Schmid-Burgk JL Hemmerling I Horvath GL Schmidt T Latz E. Cell intrinsic immunity spreads to bystander cells Via the intercellular transfer of cgamp. Nature (2013) 503(7477):530–4. doi: 10.1038/nature12640 PMC4142317 24077100

[9] Gentili M Kowal J Tkach M Satoh T Lahaye X Conrad C. Transmission of innate immune signaling by packaging of cgamp in viral particles. Science (2015) 349(6253):1232–6. doi: 10.1126/science.aab3628 26229115

[10] Bridgeman A Maelfait J Davenne T Partridge T Peng Y Mayer A. Viruses transfer the antiviral second messenger cgamp between cells. Science (2015) 349(6253):1228–32. doi: 10.1126/science.aab3632 PMC4617605 26229117

[11] Corrales L Glickman LH McWhirter SM Kanne DB Sivick KE Katibah GE. Direct activation of sting in the tumor microenvironment leads to potent and systemic tumor regression and immunity. Cell Rep (2015) 11(7):1018–30. doi: 10.1016/j.celrep.2015.04.031 PMC4440852 25959818

[12] Ritchie C Cordova AF Hess GT Bassik MC Li L. Slc19a1 is an importer of the immunotransmitter cgamp. Mol Cell (2019) 75(2):372–81.e5. doi: 10.1016/j.molcel.2019.05.006 31126740 PMC6711396

[13] Luteijn RD Zaver SA Gowen BG Wyman SK Garelis NE Onia L. Slc19a1 transports immunoreactive cyclic dinucleotides. Nature (2019) 573(7774):434–8. doi: 10.1038/s41586-019-1553-0 PMC6785039 31511694

[14] Woo S-R Fuertes Mercedes B Corrales L Spranger S Furdyna Michael J Leung Michael YK. Sting-dependent cytosolic DNA sensing mediates innate immune recognition of immunogenic tumors. Immunity (2014) 41(5):830–42. doi: 10.1016/j.immuni.2014.10.017 PMC4384884 25517615

[15] Ohkuri T Kosaka A Ishibashi K Kumai T Hirata Y Ohara K. Intratumoral administration of cgamp transiently accumulates potent macrophages for anti-tumor immunity at a mouse tumor site. Cancer Immunol Immunother (2017) 66(6):705–16. doi: 10.1007/s00262-017-1975-1 PMC11028681 28243692

[16] Ohkuri T Ghosh A Kosaka A Zhu J Ikeura M David M. Sting contributes to antiglioma immunity Via triggering type I ifn signals in the tumor microenvironment. Cancer Immunol Res (2014) 2(12):1199–208. doi: 10.1158/2326-6066.Cir-14-0099 PMC4258479 25300859

[17] Cordova AF Ritchie C Böhnert V Li L. Human Slc46a2 is the dominant cgamp importer in extracellular cgamp-sensing macrophages and monocytes. ACS Cent Sci (2021) 7(6):1073–88. doi: 10.1021/acscentsci.1c00440 PMC8228594 34235268

[18] Lahey L Mardjuki R Wen X Hess G Carroza J. RRC8A:C/E heteromeric channels are ubiquitous transporters of cGAMP. Mol Cell (2020) 80(4):578–591.e5. doi: 10.1016/j.molcel.202 PMC12629894 33171122

[19] Zhou C Chen X Planells-Cases R Chu J Wang L Cao L. Transfer of cgamp into bystander cells Via Lrrc8 volume-regulated anion channels augments sting-mediated interferon responses and anti-viral immunity. Immunity (2020) 52(5):767–81.e5. doi: 10.1016/j.immuni.2020.03.016 32277911

[20] Zhou Y Fei M Zhang G Liang WC Lin W Wu Y. Blockade of the phagocytic receptor mertk on tumor-associated macrophages enhances P2x7r-dependent sting activation by tumor-derived cgamp. Immunity (2020) 52(2):357–73.e9. doi: 10.1016/j.immuni.2020.01.014 32049051

[21] Maltbaek JH Snyder JM Stetson DB. Abcc1/Mrp1 exports cgamp and modulates cgas-dependent immunity. bioRxiv (2021) 55(10):1799–812.e4. doi: 10.1016/j.immuni.2022.08.006 PMC9561016 36070769

[22] Wei X Zhang L Yang Y Hou Y Xu Y Wang Z. Ll-37 transports immunoreactive cgamp to activate sting signaling and enhance interferon-mediated host antiviral immunity. Cell Rep (2022) 39(9):110880. doi: 10.1016/j.celrep.2022.110880 35649354

[23] Salameh A Dhein S. Pharmacology of gap junctions. new pharmacological targets for treatment of arrhythmia, seizure and cancer? Biochim Biophys Acta (BBA) - Biomembranes (2005) 1719(1):36–58. doi: 10.1016/j.bbamem.2005.09.007 16216217

[24] Müller CE Namasivayam V. Agonists, antagonists, and modulators of P2x7 receptors. In: Nicke A, editor. The P2x7 receptor: methods and protocols. New York, NY: Springer US (2022). p. 31–52. 10.1007/978-1-0716-2384-8_2 35776318

[25] Cole SPC. Multidrug resistance protein 1 (Mrp1, Abcc1), a “Multitasking” atp-binding cassette (Abc) transporter*. J Biol Chem (2014) 289(45):30880–8. doi: 10.1074/jbc.R114.609248 PMC4223294 25281745

[26] Carozza JA Böhnert V Nguyen KC Skariah G Shaw KE Brown JA. Extracellular cGAMP is a cancer-cell-produced immunotransmitter involved in radiation-induced anticancer immunity. Nat Cancer (2020) 1, 184–196. doi: 10.1038/s43018-020-0028-4 33768207 PMC7990037

[27] Li L Yin Q Kuss P Maliga Z Millan JL Wu H. Hydrolysis of 2’3’-cgamp by Enpp1 and design of nonhydrolyzable analogs. Nat Chem Biol (2014) 10(12):1043–8. doi: 10.1038/nchembio.1661 PMC4232468 25344812

[28] Belli SI van Driel IR Goding JW. Identification and characterization of a soluble form of the plasma cell membrane glycoprotein pc-1 (5’-nucleotide phosphodiesterase). Eur J Biochem (1993) 217(1):421–8. doi: 10.1111/j.1432-1033.1993.tb18261.x 8223581

[29] Zhao R Goldman ID. Folate and thiamine transporters mediated by facilitative carriers (Slc19a1-3 and Slc46a1) and folate receptors. Mol Aspects Med (2013) 34(2):373–85. doi: 10.1016/j.mam.2012.07.006 PMC3831518 23506878

[30] Zhao R Russell RG Wang Y Liu L Gao F Kneitz B. Rescue of embryonic lethality in reduced folate carrier-deficient mice by maternal folic acid supplementation reveals early neonatal failure of hematopoietic organs. J Biol Chem (2001) 276(13):10224–8. doi: 10.1074/jbc.c000905200 11266438

[31] Zhang Q Zhang X Zhu Y Sun P Zhang L Ma J. Recognition of cyclic dinucleotides and folates by human Slc19a1. Nature (2022) 612(7938):170–6. doi: 10.1038/s41586-022-05452-z 36265513

[32] Kim KY Lee G Yoon M Cho EH Park C-S Kim MG. Expression analyses revealed thymic stromal Co-Transporter/Slc46a2 is in stem cell populations and is a putative tumor suppressor. Mol Cells (2015) 38(6):548–61. doi: 10.14348/molcells.2015.0044 PMC4469913 26013383

[33] Paik D Monahan A Caffrey DR Elling R Goldman WE Silverman N. Slc46 family transporters facilitate cytosolic innate immune recognition of monomeric peptidoglycans. J Immunol (2017) 199(1):263–70. doi: 10.4049/jimmunol.1600409 PMC5513181 28539433

[34] Shin DS Min SH Russell L Zhao R Fiser A Goldman ID. Functional roles of aspartate residues of the proton-coupled folate transporter (Pcft-Slc46a1); a D156y mutation causing hereditary folate malabsorption. Blood (2010) 116(24):5162–9. doi: 10.1182/blood-2010-06-291237 PMC3012536 20805364

[35] Qiu A Jansen M Sakaris A Min SH Chattopadhyay S Tsai E. Identification of an intestinal folate transporter and the molecular basis for hereditary folate malabsorption. Cell (2006) 127(5):917–28. doi: 10.1016/j.cell.2006.09.041 17129779

[36] Dou L Chen Y-F Cowan PJ Chen X-P. Extracellular atp signaling and clinical relevance. Clin Immunol (2018) 188:67–73. doi: 10.1016/j.clim.2017.12.006 29274390

[37] Lazarowski ER. Vesicular and conductive mechanisms of nucleotide release. Purinergic Signal (2012) 8(3):359–73. doi: 10.1007/s11302-012-9304-9 PMC3360093 22528679

[38] Pellegatti P Raffaghello L Bianchi G Piccardi F Pistoia V Di Virgilio F. Increased level of extracellular atp at tumor sites: *In Vivo* imaging with plasma membrane luciferase. PloS One (2008) 3(7):e2599. doi: 10.1371/journal.pone.0002599 18612415 PMC2440522

[39] Di Virgilio F Sarti AC Falzoni S De Marchi E Adinolfi E. Extracellular atp and P2 purinergic signalling in the tumour microenvironment. Nat Rev Cancer (2018) 18(10):601–18. doi: 10.1038/s41568-018-0037-0 30006588

[40] Drill M Jones NC Hunn M O’Brien TJ Monif M. Antagonism of the atp-gated P2x7 receptor: a potential therapeutic strategy for cancer. Purinergic Signalling (2021) 17(2):215–27. doi: 10.1007/s11302-021-09776-9 PMC8155177 33728582

[41] Egan TM Khakh BS. Contribution of calcium ions to P2x channel responses. J Neurosci (2004) 24(13):3413. doi: 10.1523/JNEUROSCI.5429-03.2004 15056721 PMC6730036

[42] Gao D Li T Li XD Chen X Li QZ Wight-Carter M. Activation of cyclic gmp-amp synthase by self-DNA causes autoimmune diseases. Proc Natl Acad Sci USA (2015) 112(42):E5699–705. doi: 10.1073/pnas.1516465112 PMC4620884 26371324

[43] Pépin G De Nardo D Rootes CL Ullah TR Al-Asmari SS Balka KR. Connexin-dependent transfer of cgamp to phagocytes modulates antiviral responses. mBio (2020) 11(1):e03187–19. doi: 10.1128/mBio.03187-19 PMC6989113 31992625

[44] Bakhoum SF Ngo B Laughney AM Cavallo JA Murphy CJ Ly P. Chromosomal instability drives metastasis through a cytosolic DNA response. Nature (2018) 553(7689):467–72. doi: 10.1038/nature25432 PMC5785464 29342134

[45] Chen Q Boire A Jin X Valiente M Er EE Lopez-Soto A. Carcinoma–astrocyte gap junctions promote brain metastasis by cgamp transfer. Nature (2016) 533(7604):493–8. doi: 10.1038/nature18268 PMC5021195 27225120

[46] Green JP Swanton T Morris LV El-Sharkawy LY Cook J Yu S. Lrrc8a is essential for hypotonicity-, but not for damp-induced Nlrp3 inflammasome activation. eLife (2020) 9:e59704. doi: 10.7554/eLife.59704 33216713 PMC7679132

[47] Ritchie C Cordova AF Li L. In response to luteijn &Lt;Em<Et Al&Lt;/Em<.: concerns regarding cgamp uptake assay and evidence that Slc19a1 is not the major cgamp importer in human pbmcs. bioRxiv (2019), 798397. doi: 10.1101/798397

[48] Hou Y Wei Y Lautrup S Yang B Wang Y Cordonnier S. Nad(+) supplementation reduces neuroinflammation and cell senescence in a transgenic mouse model of alzheimer’s disease Via cgas-sting. Proc Natl Acad Sci USA (2021) 118(37):e2011226118. doi: 10.1073/pnas.2011226118 34497121 PMC8449423

[49] Yu CH Davidson S Harapas CR Hilton JB Mlodzianoski MJ Laohamonthonkul P. Tdp-43 triggers mitochondrial DNA release Via mptp to activate Cgas/Sting in als. Cell (2020) 183(3):636–49.e18. doi: 10.1016/j.cell.2020.09.020 33031745 PMC7599077

[50] Kerur N Fukuda S Banerjee D Kim Y Fu D Apicella I. Cgas drives noncanonical-inflammasome activation in age-related macular degeneration. Nat Med (2018) 24(1):50–61. doi: 10.1038/nm.4450 29176737 PMC5760363

[51] Fischer K Tschismarov R Pilz A Straubinger S Carotta S McDowell A. Cutibacterium acnes infection induces type I interferon synthesis through the cgas-sting pathway. Front Immunol (2020) 11:571334. doi: 10.3389/fimmu.2020.571334 33178195 PMC7593769

[52] King KR Aguirre AD Ye YX Sun Y Roh JD Ng RP Jr.. Irf3 and type I interferons fuel a fatal response to myocardial infarction. Nat Med (2017) 23(12):1481–7. doi: 10.1038/nm.4428 PMC6477926 29106401

[53] Cao DJ Schiattarella GG Villalobos E Jiang N May HI Li T. Cytosolic DNA sensing promotes macrophage transformation and governs myocardial ischemic injury. Circulation (2018) 137(24):2613–34. doi: 10.1161/circulationaha.117.031046 PMC5997506 29437120

[54] Yu Y Liu Y An W Song J Zhang Y Zhao X. Sting-mediated inflammation in kupffer cells contributes to progression of nonalcoholic steatohepatitis. J Clin Invest (2019) 129(2):546–55. doi: 10.1172/jci121842 PMC6355218 30561388

[55] Petrasek J Iracheta-Vellve A Csak T Satishchandran A Kodys K Kurt-Jones EA. Sting-Irf3 pathway links endoplasmic reticulum stress with hepatocyte apoptosis in early alcoholic liver disease. Proc Natl Acad Sci USA (2013) 110(41):16544–9. doi: 10.1073/pnas.1308331110 PMC3799324 24052526

[56] Iracheta-Vellve A Petrasek J Gyongyosi B Satishchandran A Lowe P Kodys K. Endoplasmic reticulum stress-induced hepatocellular death pathways mediate liver injury and fibrosis Via stimulator of interferon genes. J Biol Chem (2016) 291(52):26794–805. doi: 10.1074/jbc.M116.736991 PMC5207187 27810900

[57] Zhao Q Wei Y Pandol SJ Li L Habtezion A. Sting signaling promotes inflammation in experimental acute pancreatitis. Gastroenterology (2018) 154(6):1822–35.e2. doi: 10.1053/j.gastro.2018.01.065 29425920 PMC6112120

[58] Chung KW Dhillon P Huang S Sheng X Shrestha R Qiu C. Mitochondrial damage and activation of the sting pathway lead to renal inflammation and fibrosis. Cell Metab (2019) 30(4):784–99.e5. doi: 10.1016/j.cmet.2019.08.003 31474566 PMC7054893

[59] Ahn J Konno H Barber GN. Diverse roles of sting-dependent signaling on the development of cancer. Oncogene (2015) 34(41):5302–8. doi: 10.1038/onc.2014.457 PMC4998969 25639870

[60] Zeng L Kang R Zhu S Wang X Cao L Wang H. Alk is a therapeutic target for lethal sepsis. Sci Transl Med (2017) 9(412):eaan5689. doi: 10.1126/scitranslmed.aan5689 29046432 PMC5737927

[61] Wottawa F Bordoni D Baran N Rosenstiel P Aden K. The role of Cgas/Sting in intestinal immunity. Eur J Immunol (2021) 51(4):785–97. doi: 10.1002/eji.202048777 33577080

[62] Rodero MP Tesser A Bartok E Rice GI Della Mina E Depp M. Type I interferon-mediated autoinflammation due to dnase ii deficiency. Nat Commun (2017) 8(1):2176. doi: 10.1038/s41467-017-01932-3 29259162 PMC5736616

[63] Gall A Treuting P Elkon KB Loo YM Gale M Jr. Barber GN. Autoimmunity initiates in nonhematopoietic cells and progresses Via lymphocytes in an interferon-dependent autoimmune disease. Immunity (2012) 36(1):120–31. doi: 10.1016/j.immuni.2011.11.018 PMC3269499 22284419

[64] Di Virgilio F Giuliani AL Vultaggio-Poma V Falzoni S Sarti AC. Non-nucleotide agonists triggering P2x7 receptor activation and pore formation. Front Pharmacol (2018) 9:39. doi: 10.3389/fphar.2018.00039 29449813 PMC5799242

[65] Hernández-Carballo CY De Santiago-Castillo JA Rosales-Saavedra T Pérez-Cornejo P Arreola J. Control of volume-sensitive chloride channel inactivation by the coupled action of intracellular chloride and extracellular protons. Pflugers Arch (2010) 460(3):633–44. doi: 10.1007/s00424-010-0842-0 PMC2904426 20454973

[66] Cahalan MD Lewis RS. Role of potassium and chloride channels in volume regulation by T lymphocytes. Soc Gen Physiol Ser (1988) 43:281–301. 2479106

[67] Voss FK Ullrich F Münch J Lazarow K Lutter D Mah N. Identification of Lrrc8 heteromers as an essential component of the volume-regulated anion channel vrac. Science (2014) 344(6184):634. doi: 10.1126/science.1252826 24790029

[68] Qiu Z Dubin Adrienne E Mathur J Tu B Reddy K Miraglia Loren J. Swell1, a plasma membrane protein, is an essential component of volume-regulated anion channel. Cell (2014) 157(2):447–58. doi: 10.1016/j.cell.2014.03.024 PMC4023864 24725410

[69] Chen L König B Liu T Pervaiz S Razzaque YS Stauber T. More than just a pressure relief valve: physiological roles of volume-regulated Lrrc8 anion channels. Biol Chem (2019) 400(11):1481–96. doi: 10.1515/hsz-2019-0189 31091194

[70] Schober AL Wilson CS Mongin AA. Molecular composition and heterogeneity of the Lrrc8-containing swelling-activated osmolyte channels in primary rat astrocytes. J Physiol (2017) 595(22):6939–51. doi: 10.1113/JP275053 PMC5685816 28833202

[71] Planells-Cases R Lutter D Guyader C Gerhards NM Ullrich F Elger DA. Subunit composition of vrac channels determines substrate specificity and cellular resistance to pt-based anti-cancer drugs. EMBO J (2015) 34(24):2993–3008. doi: 10.15252/embj.201592409 26530471 PMC4687416

[72] Gaitán-Peñas H Gradogna A Laparra-Cuervo L Solsona C Fernández-Dueñas V Barrallo-Gimeno A. Investigation of Lrrc8-mediated volume-regulated anion currents in xenopus oocytes. Biophys J (2016) 111(7):1429–43. doi: 10.1016/j.bpj.2016.08.030 PMC5052465 27705766

[73] Lutter D Ullrich F Lueck JC Kempa S Jentsch TJ. Selective transport of neurotransmitters and modulators by distinct volume-regulated Lrrc8 anion channels. J Cell Sci (2017) 130(6):1122. doi: 10.1242/jcs.196253 28193731

[74] Nakamura R Numata T Kasuya G Yokoyama T Nishizawa T Kusakizako T. Cryo-em structure of the volume-regulated anion channel Lrrc8d isoform identifies features important for substrate permeation. Commun Biol (2020) 3(1):240. doi: 10.1038/s42003-020-0951-z 32415200 PMC7229184

[75] Concepcion AR Wagner LE Zhu J Tao AY Yang J Khodadadi-Jamayran A. The volume-regulated anion channel Lrrc8c suppresses T cell function by regulating cyclic dinucleotide transport and sting–P53 signaling. Nat Immunol (2022) 23(2):287–302. doi: 10.1038/s41590-021-01105-x 35105987 PMC8991407

[76] Vasiliou V Vasiliou K Nebert DW. Human atp-binding cassette (Abc) transporter family. Hum Genomics (2009) 3(3):281–90. doi: 10.1186/1479-7364-3-3-281 PMC2752038 19403462

[77] Bakos É Homolya L. Portrait of multifaceted transporter, the multidrug resistance-associated protein 1 (Mrp1/Abcc1). Pflügers Archiv - Eur J Physiol (2007) 453(5):621–41. doi: 10.1007/s00424-006-0160-8 17187268

[78] Rao VV Dahlheimer JL Bardgett ME Snyder AZ Finch RA Sartorelli AC. Choroid plexus epithelial expression of Mdr1 p glycoprotein and multidrug resistance-associated protein contribute to the blood-Cerebrospinal-Fluid drug-permeability barrier. Proc Natl Acad Sci USA (1999) 96(7):3900–5. doi: 10.1073/pnas.96.7.3900 PMC22392 10097135

[79] St-Pierre MV Stallmach T Freimoser Grundschober A Dufour JF Serrano MA Marin JJG. Temporal expression profiles of organic anion transport proteins in placenta and fetal liver of the rat. Am J Physiol-Regulatory Integr Comp Physiol (2004) 287(6):R1505–R16. doi: 10.1152/ajpregu.00279.2003 15345472

[80] Nies AT Jedlitschky G König J Herold-Mende C Steiner HH Schmitt HP. Expression and immunolocalization of the multidrug resistance proteins, Mrp1–Mrp6 (Abcc1–Abcc6), in human brain. Neuroscience (2004) 129(2):349–60. doi: 10.1016/j.neuroscience.2004.07.051 15501592

[81] Maltbaek JH Cambier S Snyder JM Stetson DB. Abcc1 transporter exports the immunostimulatory cyclic dinucleotide cgamp. Immunity (2022) 55(10):1799–812.e4. doi: 10.1016/j.immuni.2022.08.006 36070769 PMC9561016

[82] Stetson DB Ko JS Heidmann T Medzhitov R. Trex1 prevents cell-intrinsic initiation of autoimmunity. Cell (2008) 134(4):587–98. doi: 10.1016/j.cell.2008.06.032 PMC2626626 18724932

[83] Gray EE Treuting PM Woodward JJ Stetson DB. Cutting edge: cgas is required for lethal autoimmune disease in the Trex1-deficient mouse model of aicardi-goutières syndrome. J Immunol (2015) 195(5):1939–43. doi: 10.4049/jimmunol.1500969 PMC4546858 26223655

[84] Takabe K Kim RH Allegood JC Mitra P Ramachandran S Nagahashi M. Estradiol induces export of sphingosine 1-phosphate from breast cancer cells Via Abcc1 and Abcg2. J Biol Chem (2010) 285(14):10477–86. doi: 10.1074/jbc.M109.064162 PMC2856255 20110355

[85] Zhang Q-Y Yan Z-B Meng Y-M Hong X-Y Shao G Ma J-J. Antimicrobial peptides: mechanism of action, activity and clinical potential. Military Med Res (2021) 8(1):48. doi: 10.1186/s40779-021-00343-2 PMC8425997 34496967

[86] Xhindoli D Pacor S Benincasa M Scocchi M Gennaro R Tossi A. The human cathelicidin ll-37 — a pore-forming antibacterial peptide and host-cell modulator. Biochim Biophys Acta (BBA) - Biomembranes (2016) 1858(3):546–66. doi: 10.1016/j.bbamem.2015.11.003 26556394

[87] Vandamme D Landuyt B Luyten W Schoofs L. A comprehensive summary of ll-37, the factotum human cathelicidin peptide. Cell Immunol (2012) 280(1):22–35. doi: 10.1016/j.cellimm.2012.11.009 23246832

[88] Yamasaki K Schauber J Coda A Lin H Dorschner RA Schechter NM. Kallikrein-mediated proteolysis regulates the antimicrobial effects of cathelicidins in skin. FASEB J (2006) 20(12):2068–80. doi: 10.1096/fj.06-6075com 17012259

[89] Sørensen OE Follin P Johnsen AH Calafat J Tjabringa GS Hiemstra PS. Human cathelicidin, hcap-18, is processed to the antimicrobial peptide ll-37 by extracellular cleavage with proteinase 3. Blood (2001) 97(12):3951–9. doi: 10.1182/blood.v97.12.3951 11389039

[90] Turner J Cho Y Dinh NN Waring AJ Lehrer RI. Activities of ll-37, a cathelin-associated antimicrobial peptide of human neutrophils. Antimicrob Agents Chemother (1998) 42(9):2206–14. doi: 10.1128/aac.42.9.2206 PMC105778 9736536

[91] Lee CC Sun Y Qian S Huang HW. Transmembrane pores formed by human antimicrobial peptide ll-37. Biophys J (2011) 100(7):1688–96. doi: 10.1016/j.bpj.2011.02.018 PMC3072607 21463582

[92] Sancho-Vaello E Gil-Carton D François P Bonetti E-J Kreir M Pothula KR. The structure of the antimicrobial human cathelicidin ll-37 shows oligomerization and channel formation in the presence of membrane mimics. Sci Rep (2020) 10(1):17356. doi: 10.1038/s41598-020-74401-5 33060695 PMC7562864

[93] Ding B Soblosky L Nguyen K Geng J Yu X Ramamoorthy A. Physiologically-relevant modes of membrane interactions by the human antimicrobial peptide, ll-37, revealed by sfg experiments. Sci Rep (2013) 3(1):1854. doi: 10.1038/srep01854 23676762 PMC3655398

[94] Yang B Good D Mosaiab T Liu W Ni G Kaur J. Significance of ll-37 on immunomodulation and disease outcome. BioMed Res Int (2020) 2020:8349712. doi: 10.1155/2020/8349712 32509872 PMC7246396

[95] Yoon S-H Hwang I Lee E Cho H-J Ryu JH Kim T-G. Antimicrobial peptide ll-37 drives rosacea-like skin inflammation in an Nlrp3-dependent manner. J Invest Dermatol (2021) 141(12):2885–94.e5. doi: 10.1016/j.jid.2021.02.745 33745908

[96] Chamilos G Gregorio J Meller S Lande R Kontoyiannis DP Modlin RL. Cytosolic sensing of extracellular self-DNA transported into monocytes by the antimicrobial peptide Ll37. Blood (2012) 120(18):3699–707. doi: 10.1182/blood-2012-01-401364 PMC3488884 22927244

[97] Lande R Gregorio J Facchinetti V Chatterjee B Wang Y-H Homey B. Plasmacytoid dendritic cells sense self-DNA coupled with antimicrobial peptide. Nature (2007) 449(7162):564–9. doi: 10.1038/nature06116 17873860

[98] Sandgren S Wittrup A Cheng F Jönsson M Eklund E Busch S. The human antimicrobial peptide ll-37 transfers extracellular DNA plasmid to the nuclear compartment of mammalian cells Via lipid rafts and proteoglycan-dependent endocytosis*. J Biol Chem (2004) 279(17):17951–6. doi: 10.1074/jbc.M311440200 14963039

[99] Kirchhoff C Cypionka H. Propidium ion enters viable cells with high membrane potential during live-dead staining. J Microbiol Methods (2017) 142:79–82. doi: 10.1016/j.mimet.2017.09.011 28927972

[100] Totland MZ Rasmussen NL Knudsen LM Leithe E. Regulation of gap junction intercellular communication by connexin ubiquitination: physiological and pathophysiological implications. Cell Mol Life Sci (2020) 77(4):573–91. doi: 10.1007/s00018-019-03285-0 PMC7040059 31501970

[101] Laird DW. Life cycle of connexins in health and disease. Biochem J (2006) 394(Pt 3):527–43. doi: 10.1042/BJ20051922 PMC1383703 16492141

[102] Vinken M. Introduction: connexins, pannexins and their channels as gatekeepers of organ physiology. Cell Mol Life Sci (2015) 72(15):2775–8. doi: 10.1007/s00018-015-1958-3 PMC4562364 26084871

[103] Patel SJ King KR Casali M Yarmush ML. DNA-Triggered innate immune responses are propagated by gap junction communication. Proc Natl Acad Sci (2009) 106(31):12867–72. doi: 10.1073/pnas.0809292106 PMC2722330 19617563

[104] Wang J Li P Yu Y Fu Y Jiang H Lu M. Pulmonary surfactant-biomimetic nanoparticles potentiate heterosubtypic influenza immunity. Science (2020) 367(6480):eaau0810. doi: 10.1126/science.aau0810 32079747 PMC7432993

[105] Gossman DG Zhao H-B. Hemichannel-mediated inositol 1,4,5-trisphosphate (Ip3) release in the cochlea: a novel mechanism of Ip3 intercellular signaling. Cell Commun Adhes (2008) 15(4):305–15. doi: 10.1080/15419060802357217 PMC5543712 18979296

[106] Valiunas V. Cyclic nucleotide permeability through unopposed connexin hemichannels. Front Pharmacol (2013) 4:75. doi: 10.3389/fphar.2013.00075 23760880 PMC3674318

[107] Loiselle AE Jiang JX Donahue HJ. Gap junction and hemichannel functions in osteocytes. Bone (2013) 54(2):205–12. doi: 10.1016/j.bone.2012.08.132 23069374

[108] Maxwell KL Frappier L. Viral proteomics. Microbiol Mol Biol Rev (2007) 71(2):398–411. doi: 10.1128/mmbr.00042-06 17554050 PMC1899879

[109] Yáñez-Mó M Siljander PR Andreu Z Zavec AB Borràs FE Buzas EI. Biological properties of extracellular vesicles and their physiological functions. J Extracell Vesicles (2015) 4:27066. doi: 10.3402/jev.v4.27066 25979354 PMC4433489

[110] Burgos-Ravanal R Campos A Díaz-Vesga MC González MF León D Lobos-González L. Extracellular vesicles as mediators of cancer disease and as nanosystems in theranostic applications. Cancers (Basel) (2021) 13(13):3324. doi: 10.3390/cancers13133324 34283059 PMC8268753

[111] McAndrews KM Che SPY LeBleu VS Kalluri R. Effective delivery of sting agonist using exosomes suppresses tumor growth and enhances antitumor immunity. J Biol Chem (2021) 296:100523. doi: 10.1016/j.jbc.2021.100523 33711340 PMC8042450

[112] Skopelja-Gardner S An J Elkon KB. Role of the cgas–sting pathway in systemic and organ-specific diseases. Nat Rev Nephrol (2022) 18(9):558–72. doi: 10.1038/s41581-022-00589-6 PMC9214686 35732833

[113] Harding SM Benci JL Irianto J Discher DE Minn AJ Greenberg RA. Mitotic progression following DNA damage enables pattern recognition within micronuclei. Nature (2017) 548(7668):466–70. doi: 10.1038/nature23470 PMC5857357 28759889

[114] Mackenzie KJ Carroll P Martin CA Murina O Fluteau A Simpson DJ. Cgas surveillance of micronuclei links genome instability to innate immunity. Nature (2017) 548(7668):461–5. doi: 10.1038/nature23449 PMC5870830 28738408

[115] Marcus A Mao AJ Lensink-Vasan M Wang L Vance RE Raulet DH. Tumor-derived cgamp triggers a sting-mediated interferon response in non-tumor cells to activate the nk cell response. Immunity (2018) 49(4):754–63.e4. doi: 10.1016/j.immuni.2018.09.016 30332631 PMC6488306

[116] Schadt L Sparano C Schweiger NA Silina K Cecconi V Lucchiari G. Cancer-Cell-Intrinsic cgas expression mediates tumor immunogenicity. Cell Rep (2019) 29(5):1236–48.e7. doi: 10.1016/j.celrep.2019.09.065 31665636

[117] Li J Duran MA Dhanota N Chatila WK Bettigole SE Kwon J. Metastasis and immune evasion from extracellular cgamp hydrolysis. Cancer Discov (2021) 11(5):1212–27. doi: 10.1158/2159-8290.Cd-20-0387 PMC8102348 33372007

[118] Bakhoum SF Kabeche L Wood MD Laucius CD Qu D Laughney AM. Numerical chromosomal instability mediates susceptibility to radiation treatment. Nat Commun (2015) 6:5990. doi: 10.1038/ncomms6990 25606712 PMC4516720

[119] Dou Z Ghosh K Vizioli MG Zhu J Sen P Wangensteen KJ. Cytoplasmic chromatin triggers inflammation in senescence and cancer. Nature (2017) 550(7676):402–6. doi: 10.1038/nature24050 PMC5850938 28976970

[120] Crunchbase. angarus therapeutics (2022). Available at: https://www.crunchbase.com/organization/angarus-therapeutics.

[121] Meric-Bernstam F Sweis RF Hodi FS Messersmith WA Andtbacka RHI Ingham M. Phase I dose-escalation trial of Miw815 (Adu-S100), an intratumoral sting agonist, in patients with Advanced/Metastatic solid tumors or lymphomas. Clin Cancer Res (2021) 28(4):677–88. doi: 10.1158/1078-0432.CCR-21-1963 34716197

[122] Van Herck S Feng B Tang L. Delivery of sting agonists for adjuvanting subunit vaccines. Advanced Drug Delivery Rev (2021) 179:114020. doi: 10.1016/j.addr.2021.114020 34756942

[123] Garland KM Sheehy TL Wilson JT. Chemical and biomolecular strategies for sting pathway activation in cancer immunotherapy. Chem Rev (2022) 122(6):5977–6039. doi: 10.1021/acs.chemrev.1c00750 35107989 PMC8994686

[124] Ozasa K Temizoz B Kusakabe T Kobari S Momota M Coban C. Cyclic gmp-amp triggers asthma in an il-33-Dependent manner that is blocked by amlexanox, a Tbk1 inhibitor. Front Immunol (2019) 10:2212. doi: 10.3389/fimmu.2019.02212 31616416 PMC6775192

[125] Van Noort A Nelsen A Pillatzki AE Diel DG Li F Nelson E. Intranasal immunization of pigs with porcine reproductive and respiratory syndrome virus-like particles plus 2’, 3’-cgamp vaccigrade™ adjuvant exacerbates viremia after virus challenge. Virol J (2017) 14(1):76. doi: 10.1186/s12985-017-0746-0 28403874 PMC5389191

[126] Varma DM Batty CJ Stiepel RT Graham-Gurysh EG Roque JA 3rd Pena ES. Development of an intranasal gel for the delivery of a broadly acting subunit influenza vaccine. ACS Biomater Sci Eng (2022) 8(4):1573–82. doi: 10.1021/acsbiomaterials.2c00015 PMC9627116 35353486

[127] Luo J Liu XP Xiong FF Gao FX Yi YL Zhang M. Enhancing immune response and heterosubtypic protection ability of inactivated H7n9 vaccine by using sting agonist as a mucosal adjuvant. Front Immunol (2019) 10:2274. doi: 10.3389/fimmu.2019.02274 31611875 PMC6777483

[128] Takaki H Takashima K Oshiumi H Ainai A Suzuki T Hasegawa H. Cgamp promotes germinal center formation and production of iga in nasal-associated lymphoid tissue. Med Sci (Basel) (2017) 5(4):35. doi: 10.3390/medsci5040035 29258267 PMC5753664

[129] Gutjahr A Papagno L Nicoli F Kanuma T Kuse N Cabral-Piccin MP. The sting ligand cgamp potentiates the efficacy of vaccine-induced Cd8+ T cells. JCI Insight (2019) 4(7):e125107. doi: 10.1172/jci.insight.125107 30944257 PMC6483644

[130] Wang J Li P Wu MX. Natural sting agonist as an “Ideal” adjuvant for cutaneous vaccination. J Invest Dermatol (2016) 136(11):2183–91. doi: 10.1016/j.jid.2016.05.105 PMC6091668 27287182

[131] Junkins RD Gallovic MD Johnson BM Collier MA Watkins-Schulz R Cheng N. A robust microparticle platform for a sting-targeted adjuvant that enhances both humoral and cellular immunity during vaccination. J Control Release (2018) 270:1–13. doi: 10.1016/j.jconrel.2017.11.030 29170142 PMC5808851

[132] Zhu W Wei L Dong C Wang Y Kim J Ma Y. Cgamp-adjuvanted multivalent influenza mrna vaccines induce broadly protective immunity through cutaneous vaccination in mice. Mol Ther Nucleic Acids (2022) 30:421–37. doi: 10.1016/j.omtn.2022.10.024 PMC9668623 36420215

[133] Chauveau L Bridgeman A Tan TK Beveridge R Frost JN Rijal P. Inclusion of cGAMP within virus-like particle vaccines enhances their immunogenicity. EMBO Reports (2021) 22:e52447. doi: 10.1101/2020.01.03.893586 34142428 PMC8339669

[134] Jneid B Bochnakian A Hoffmann C Delisle F Djacoto E Sirven P. Selective sting stimulation in dendritic cells primes antitumor T cell responses. Sci Immunol (2023) 8(79):eabn6612. doi: 10.1126/sciimmunol.abn6612 36638189

[135] Vassilieva EV Li S Korniychuk H Taylor DM Wang S Prausnitz MR. Cgamp/Saponin adjuvant combination improves protective response to influenza vaccination by microneedle patch in an aged mouse model. Front Immunol (2020) 11:583251. doi: 10.3389/fimmu.2020.583251 33603732 PMC7884748

[136] Vassilieva EV Taylor DW Compans RW. Combination of sting pathway agonist with saponin is an effective adjuvant in immunosenescent mice. Front Immunol (2019) 10:3006. doi: 10.3389/fimmu.2019.03006 31921219 PMC6935580

[137] Borriello F Pietrasanta C Lai JCY Walsh LM Sharma P O’Driscoll DN. Identification and characterization of stimulator of interferon genes as a robust adjuvant target for early life immunization. Front Immunol (2017) 8:1772. doi: 10.3389/fimmu.2017.01772 29312305 PMC5732947

[138] Liu N Pang X Zhang H Ji P. The cgas-sting pathway in bacterial infection and bacterial immunity. Front Immunol (2021) 12:814709. doi: 10.3389/fimmu.2021.814709 35095914 PMC8793285

[139] Zhang Y Yeruva L Marinov A Prantner D Wyrick PB Lupashin V. The DNA sensor, cyclic gmp-amp synthase, is essential for induction of ifn-B during chlamydia trachomatis infection. J Immunol (2014) 193(5):2394–404. doi: 10.4049/jimmunol.1302718 PMC4212656 25070851

[140] Carozza JA Cordova AF Brown JA AlSaif Y Böhnert V Cao X. Enpp1'S regulation of extracellular cgamp is a ubiquitous mechanism of attenuating sting signaling. Proc Natl Acad Sci (2022) 119(21):e2119189119. doi: 10.1073/pnas.2119189119 35588451 PMC9173814

[141] Eaglesham JB Pan Y Kupper TS Kranzusch PJ. Viral and metazoan poxins are cgamp-specific nucleases that restrict cgas-sting signalling. Nature (2019) 566(7743):259–63. doi: 10.1038/s41586-019-0928-6 PMC6640140 30728498

[142] Dodantenna N Ranathunga L Chathuranga WAG Weerawardhana A Cha J-W Subasinghe A. African Swine fever virus Ep364r and C129r target cyclic gmp-amp to inhibit the cgas-sting signaling pathway. J Virol (2022) 96(15):e0102222–e. doi: 10.1128/jvi.01022-22 PMC9364804 35861515

[143] Uggenti C Lepelley A Depp M Badrock AP Rodero MP El-Daher MT. Cgas-mediated induction of type I interferon due to inborn errors of histone pre-mrna processing. Nat Genet (2020) 52(12):1364–72. doi: 10.1038/s41588-020-00737-3 33230297

[144] Luther J Khan S Gala MK Kedrin D Sridharan G Goodman RP. Hepatic gap junctions amplify alcohol liver injury by propagating cgas-mediated Irf3 activation. Proc Natl Acad Sci USA (2020) 117(21):11667–73. doi: 10.1073/pnas.1911870117 PMC7261084 32393626

[145] Kolbrink B Riebeling T Kunzendorf U Krautwald S. Plasma membrane pores drive inflammatory cell death. Front Cell Dev Biol (2020) 8:817. doi: 10.3389/fcell.2020.00817 32974349 PMC7471660

[146] Osińska I Popko K Demkow U. Perforin: an important player in immune response. Cent Eur J Immunol (2014) 39(1):109–15. doi: 10.5114/ceji.2014.42135 PMC4439970 26155110

[147] Kayagaki N Kornfeld OS Lee BL Stowe IB O’Rourke K Li Q. Ninj1 mediates plasma membrane rupture during lytic cell death. Nature (2021) 591(7848):131–6. doi: 10.1038/s41586-021-03218-7 33472215

[148] Hu J Wang X Xing Y Rong E Ning M Smith J. Origin and development of oligoadenylate synthetase immune system. BMC Evol Biol (2018) 18(1):201. doi: 10.1186/s12862-018-1315-x 30587119 PMC6307210

[149] Liu X Liu Z Wu Z Ren J Fan Y Sun L. Resurrection of endogenous retroviruses during aging reinforces senescence. Cell (2023) 186(2):287–304.e26. doi: 10.1016/j.cell.2022.12.017 36610399

[150] Pastuzyn ED Day CE Kearns RB Kyrke-Smith M Taibi AV McCormick J. The neuronal gene arc encodes a repurposed retrotransposon gag protein that mediates intercellular rna transfer. Cell (2018) 172(1-2):275–88.e18. doi: 10.1016/j.cell.2017.12.024 29328916 PMC5884693

[151] Segel M Lash B Song J Ladha A Liu CC Jin X. Mammalian retrovirus-like protein Peg10 packages its own mrna and can be pseudotyped for mrna delivery. Science (2021) 373(6557):882–9. doi: 10.1126/science.abg6155 PMC8431961 34413232

[152] Breitinger U Farag NS Sticht H Breitinger H-G. Viroporins: structure, function, and their role in the life cycle of sars-Cov-2. Int J Biochem Cell Biol (2022) 145:106185–. doi: 10.1016/j.biocel.2022.106185 PMC8868010 35219876

